# Effects of ionization on stability of 1-methylcytosine — DFT and PCM studies

**DOI:** 10.1007/s00894-016-3020-2

**Published:** 2016-06-03

**Authors:** Ewa D. Raczyńska, Piotr Michalec, Marcin Zalewski, Mariusz Sapuła

**Affiliations:** Department of Chemistry, Warsaw University of Life Sciences (SGGW), 02-776 Warszawa, Poland; Faculty of Agriculture and Biology, SGGW, 02-776 Warszawa, Poland; Interdisciplinay Department of Biotechnology, SGGW, 02-776 Warszawa, Poland

**Keywords:** Acid-base parameters, Delocalization of n- and π-electrons, Effects of ionization, 1-Methylcytosine, Prototropic tautomers and zwitterions, Tautomeric conversions

## Abstract

Consequences of ionization were studied by quantum-chemical methods (DFT and PCM) for 1-methylcytosine (**MC**)—a model of the nucleobase cytosine (**C**) connected with sugar in DNA. For calculations, three prototropic tautomers (one amino and two imino forms) and two imino zwitterions were considered, including conformational or configurational isomerism of *exo* heterogroups. Ionization and interactions between neighboring groups affect intramolecular proton-transfers, geometric and thermodynamic parameters, and electron delocalization for individual isomers. We discovered that an imino isomer is present in the isomeric mixture in the highest amount for positively ionized **MC**. Its contribution in neutral and negatively ionized **MC** is considerably smaller. Acid-base parameters for selected radical ions were estimated in the gas phase and compared to those of neutral **MC**. Gas-phase acidity of radical cations is close to that of the conjugate acid of **MC**, and gas-phase basicity of radical anions is close to that of the conjugate base of **MC**. Various routes of amino-imino conversion between neutral and ionized isomers were considered. Energetic-barrier for intramolecular proton-transfer in **MC** is close to that in the parent system—formamidine.

**Electronic supplementary material:**

The online version of this article (doi:10.1007/s00894-016-3020-2) contains supplementary material, which is available to authorized users.

## Introduction

Ionization reactions, in which a neutral molecule (M) changes its oxidation state and transforms into charged radicals, radical cation (M – e → M^+•^) or radical anion (M + e → M^-•^), are well recognized in chemistry [1, 2]. A large number of documents can be found in the literature for simple inorganic and organic species, as well as for more complex biomolecules. Energetic parameters, called the “ionization potential” (IP) or “ionization energy” (IE) for positive ionization and the “electron affinity” (EA) for negative ionization, have been experimentally determined in the gas phase for about a thousand compounds and compiled in the NIST Chemistry Web Book, available online since 1996 [3].

Ionized forms, radical cations and radical anions, can be generated in the presence of ionizing agents, *e.g*., electrons, atoms, ions, *etc*. They can be identified in various types of mass spectrometers during positive or negative ionization [3–6]. They can also be detected by other spectroscopic techniques such as zero kinetic energy photoelectron spectroscopy [7], infrared depletion spectroscopy [8, 9], or time-resolved resonance Raman spectroscopy combined with pulse radiolysis [10–14]. Ionization reactions and mechanisms of one-electron loss or one-electron gain can be analyzed by quantum-chemical methods, applied to isolated (in *vacuo*), as well as to solvated molecules [15–21]. For simple molecules, the IP (or IE) and EA values are associated with an atom or group which loses or gains one-electron [15]. For more complex biomolecules, for which prototropic tautomerism coupled with resonance very often takes place, isomers favored for neutral forms not always predominate for charged radicals [22–24], and it is difficult to indicate the ionization site from simple experiments. To understand the ionization processes and to explain the mechanisms of ionization reactions for tautomeric systems, quantum-chemical calculations should be performed for complete tautomeric mixtures of neutral and ionized forms, and physicochemical properties analyzed in detail.

For free nucleobases, uracil (U), thymine (T), cytosine (C), adenine (A), guanine (G), and for their model compounds, imidazole, purine, hydroxy- and amino-azines, the complete tautomeric mixtures in the gas phase have already been investigated [23–28]. One-electron loss and one-electron gain change acid-base properties of tautomeric groups, and consequently, change compositions of tautomeric mixtures and properties of nucleobases. These effects shed light on chemical changes in DNA, affecting aging processes, as well as various diseases, tumors, and cancers.

**Fig. 1:**
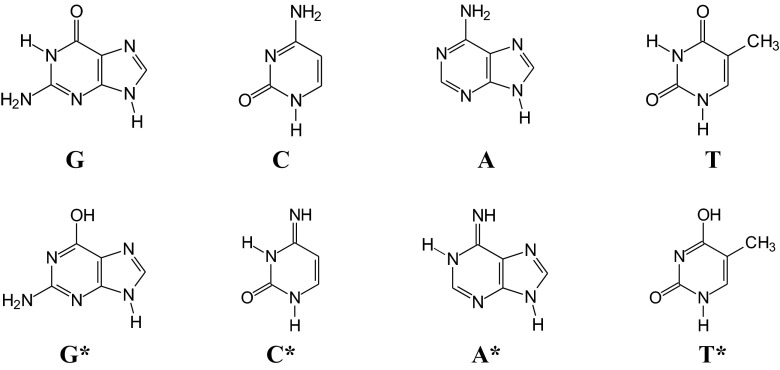
Canonical (**G**, **C**, **A**, and **T**) and rare forms (G*, C*, A*, and T*) of nucleobases

The phenomenon of prototropic tautomerism in heterocycles was already well known in the 1950s [29], when the molecular structure of nucleic acids was proposed by Watson and Crick [30]. The authors suggested that point mutations can occur in nucleic acids when canonical tautomeric forms of nucleobases change into their rare forms [31]. In normal nucleic acids, nucleobases have their canonical forms: amide and amino forms. Nevertheless, their rare tautomers, iminol, and imino forms are also possible (Fig. 1). In normal DNA, cytosine (**C**) is paired with guanine (**G**), and thymine (**T**) is paired with adenine (**A**) [30]. However, the pairing can be impossible when the tautomeric preferences change. For example, the rare isomer of cytosine can be paired with adenine, and during DNA replication it can be replaced by thymine leading to the **GC** → **AT** transition [31]. Mispairs can also take place when one of the bases is ionized [32]. This type of mispair seems to occur most frequently than the mispairs being a consequence of neutral rare forms.

DNA mutations were theoretically modeled by Löwdin in the 1960s [33, 34]. Taking the Watson and Crick hypothesis into account, the author proposed a model in which a double intermolecular proton-transfer in base pair **GC** or **AT** is possible. This proton-transfer can change the canonical forms of nucleobases into their rare forms. Consequently, after multiple DNA replications the **GC** pair can be substituted by the **AT** pair and *vice versa*. This pioneering model of double intermolecular proton-transfer for neutral and ionized pairs has been extensively studied by various quantum-chemical methods in the last 20 years. Some representative reports are cited here [32, 35–55]. Kumar and Sevilla [32], reviewing changes occurring in DNA exposed to high-energy radiation, paid attention to very fast (<20 ns) proton-coupled electron-transfer and discussed numerous experimental and theoretical works on intermolecular proton-transfer for radical ions. The authors found that in frozen aqueous solution upon one-electron loss a nucleobase becomes more acidic and can be deprotonated by a base, and upon one-electron gain it becomes more basic and can be protonated by an acid [56].

However, in the gas phase and in less polar environments, such as lipids and proteins present in living organisms, acid-base properties and proton-transfer reactions can be different from those in aqueous solution. For example, in aqueous solution amino acids and peptides exist in their zwitterionic forms, whereas in the gas phase intramolecular proton-transfers from acidic to basic groups for amphiprotic compounds are prohibited (Δ*G* > 100 kcal mol^-1^) [57]. Another example is guanidine which is a very strong base in aqueous solution (p*K*_a_ > 13), whereas in the gas phase its basicity is lower than that of triethylamine [3, 58]. Hence, it is possible that acid-base properties of radical ions, their stabilities and reactivities can be different in the gas phase than in aqueous solution.

Some information on stability of gaseous radicals of nucleobases can be found in reports of Tureček [5, 6], who used MS technologies. Nevertheless, there are little data on their acid-base properties. More information can be found for hydrated radicals of nucleobases [12–14, 32, 56]. For example, Sevilla and co-workers [56] proposed singly (pH 7-9) and doubly (pH > 11) deprotonated species for the guanine canonical radical cation in frozen aqueous solution, whereas Choi et al. [12] showed that the deprotonated guanine radical cation can be rapidly converted by protonation to a new radical cation, its tautomer. The authors employed different spectroscopic techniques. Sevilla and co-workers used ESR and UV-visible spectroscopy, whereas Choi et al. applied time-resolved resonance Raman spectroscopy combined with pulse radiolysis. The phenomena observed by Sevilla, Choi, and their co-workers clearly showed that proton-transfer reactions for ionized nucleobases, not yet paired in the DNA structure, may be crucial for better understanding various aging and disease processes. These fascinating experiments and interesting discussions between the authors [13, 14], encouraged us to continue our studies on free nucleobases and their models, undertaken about ten years ago.

**Chart 1:**
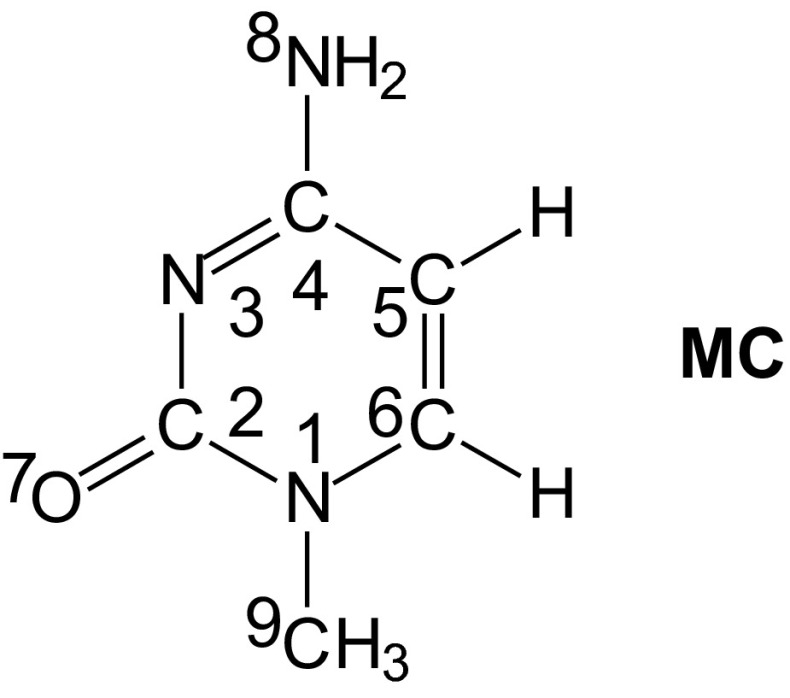
Structure of 1-methylcytosine (**MC**)

In this work, we chose 1-methylcytosine (**MC** in Chart 1) for modeling some tendencies of cytosine, combined with sugar in the DNA structure, to intramolecular proton-transfers. **MC** is frequently chosen to understand various properties of cytosine included in nucleic acids [59–64], because its substitution at N1 eliminates one labile proton and reduces a large number of prototropic tautomers occurring for isolated cytosine [28] to that possible for cytosine connected with sugar. This change can induce significant differences in geometric parameters and in relative stabilities of canonical and rare forms of cytosine. For our investigations, the neutral (**MC**), positively ionized (**MC** – e → **MC**^+•^), and negatively ionized forms (**MC** + e → **MC**^-•^) were taken into account.

We studied the effects of positive and negative ionization on **MC** isomers in two extreme environments, the gas phase and aqueous solution. Two levels of theory were chosen here: the density functional theory (DFT) method [65] with the three-parameter hybrid functional of Becke [66] and the non-local correlation functional of Lee, Yang, and Parr (B3LYP) [67], and the 6-311+G(d,p) basis set [68], and the polarizable continuum model (PCM) [69, 70]. For selected isomers, we also tested the aug-cc-pVDZ basis set [71]. The PCM model does not include typical specific interactions with water molecules. Nevertheless, the PCM results can provide information on medium-polarity effects. The B3LYP functional was recommended and applied by Schaefer and co-workers for charged forms, particularly for radical anions [15, 20, 21, 50, 51], and used by Sevilla and co-workers for ionized nucleobases [52, 53, 56]. It has been successfully applied to proton-transfer reactions for mono- and polyfunctional bases [72, 73] as well as to tautomeric conversions for simple [74–82] and more complex tautomeric systems including ionized nucleobases and their model compounds [24–28]. The PCM method was already used for investigations of radical ions of DNA bases and also for estimations of the IPs and/or EAs in aqueous solution [19, 83–85]. More details on selection of quantum-chemical methods are given in Electronic supplementary material (ESM).

## Methods

**Fig. 2:**
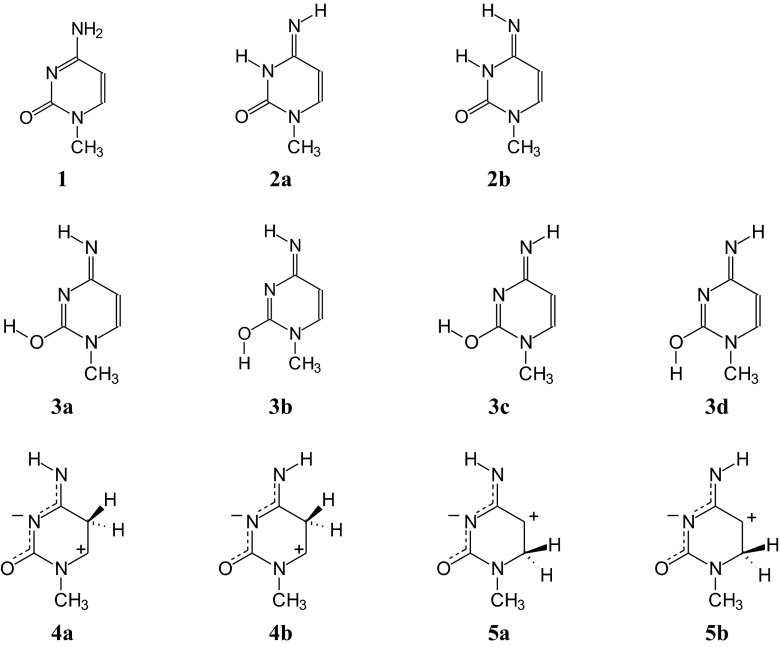
Eleven isomers considered here for neutral and ionized 1-methylcytosine

Geometries of neutral and ionized forms of 1-methylcytosine (Fig. 2) were optimized at two levels of theory {B3LYP/6-311+G(d,p) [65–68] and PCM(water)//B3LYP/6-311+G(d,p) [69, 70]} using the Gaussian-03 program [86]. For selected radical anions, calculations were also carried out at the B3LYP/aug-cc-pVDZ level [65–67, 71]. Thermodynamic parameters were estimated at the same level of theory which was applied to geometry optimization. Computational details are given in ESM. The geometry-based harmonic oscillator model of electron delocalization (HOMED) indices [87, 88] were estimated for neutral and ionized isomers of **MC** using the same parameterization as that described previously [28, 88]. Details on the HOMED estimation are given in ESM. The harmonic oscillator model of aromaticity (HOMA) index [89–91], and the harmonic oscillator model of heterocyclic electron delocalization (HOMHED) index [92] were not applied here for **MC** for the reasons discussed previously [24, 26].

## Results and discussion

### Possible isomers for MC

Similar to the canonical form of cytosine included in DNA, **MC** possesses one proton at N8 that can be intramolecularly transferred between three tautomeric atoms, N8, O7, and N3. Each proton-transfer is accompanied by migration of double bond(s) [29]. The complete tautomeric mixture for **MC** consists of three prototropic tautomers (**1**-**3**) (Fig. 2). Gutowski and co-workers [23, 93], characterizing the valence anionic states of **MC**, considered additionally two zwitterionic forms (**4a** and **5a**) with the proton at C5 and C6. Rotational and/or geometric isomerism for *exo* groups (–OH and = NH, respectively) are also possible. Hence, considering these four types of isomerism: prototropic tautomerism, formation of zwitterions, rotational and geometric isomerism, a mixture of 11 isomers of **MC** should be analyzed to obtain a complete picture of intramolecular proton-transfer. For neutral **MC**, zwitterionic forms probably do not exist. However, they can be stabilized by ions or free electrons [93]. They can occur for ionized (redox) forms. Analogous to cytosine, present in normal DNA, the amino isomer **1** of **MC** can be called the “canonical” tautomer [30], and the isomer **2a**, responsible for DNA mutations, can be named the “rare” form [31].

The mixture of all 11 **MC** isomers can only be investigated by quantum-chemical methods. Experiments give a possibility to characterize the major forms [94]. Rare isomers, and sometimes even minor ones, are undetectable for tautomeric systems. Very often, their signals, being in the background, cannot be identified. Some isomers of neutral 1-methylcytosine have already been studied in various environments [95–101]. There are also a few documents on radicals [102–107] and metal complexes [108–110]. However, literature data are not complete for **MC** and only a little information can be derived on ionization effects. Even for the neutral forms, conformational or configurational isomerism of the *exo* groups has not always been taken into account.

### Geometries, charges, spin densities, and delocalization of n- and π-electrons

DFT calculations showed that heavy atoms of neutral **MC** isomers are in the ring plane (Table S1 in ESM) indicating that n- and π-electrons are well delocalized. For the amino tautomer **1**, its *exo* NH_2_ group has a pyramidal conformation analogous to that in adenine and other amino derivatives of pyrimidine [24, 26, 81]. For the imino isomers **2a**-**3d**, proton-transfer from N8 to N3 or O7 does not destroy the ring planarity. The *exo* groups are in the ring plane. The CC and CX (X = N or O) bond lengths depend on position of labile proton and on isomerism of *exo* groups. Ionization affects the CC and CX bond lengths and the ring planarity. Similar to isolated cytosine [28], the *exo* NH_2_ group is in the ring plane for **1**^+•^, while this group has a twisted pyramidal conformation for **1**^-•^. The *exo* =NH and −OH groups are more twisted for radical anions than for radical cations. However, the dihedral angle is not larger than 12°.

An analysis of charge and total atomic spin densities (Table S2 in ESM) shows important differences in ionization mechanisms. One electron can be taken from different heteroatoms and/or π-bonds in **MC** isomers. Various sites in **MC** isomers can also attach one electron. Generally, the labile proton position (N8, N3, O7, C5, or C6) generates the charge distribution and unpaired spin density on other atoms. For **MC**^+•^ isomers, the spin density is delocalized on the following atoms: N1 (for **1**-**3d**), N3 (for **1** and **3a**-**5b**), N9 (for **2a**-**5b**), O7 (for **1**, **4a** and **4b**), and C5 (for **1**-**3d**, **5a**, and **5b**). For **MC**^-•^ isomers, the spin density is mainly carried by carbon atoms: C2 (for **3a**-**3d**), C4 (for **1**-**3d**), C5 (for **3a**-**3d**, **5a**, and **5b**), and C6 (for **1**-**2b**, **4a**, and **4b**), but its high concentration also exists on N3 (for **1**) and N9 (for **2a** and **2b**). Detailed analysis showed clearly that the unpaired electron stabilizes the structures **4a**, **4b**, **5a**, and **5b**, which were not found for neutral **MC** as zwitterions.

Delocalization of n- and π-electrons in neutral and ionized **MC** isomers can be quantitatively measured using the geometry-based HOMED indices. Estimations were made for geometries optimized at the DFT level for the ring (six bonds – HOMED6) and for the whole tautomeric system, including *exo* groups (eight bonds – HOMED8). The calculated HOMED6 and HOMED8 values are given in Table S3 (ESM). For the neutral canonical **MC**-isomer **1**, n-π conjugation in the six-membered ring (HOMED6 = 0.768) is analogous to that for the corresponding isomer of cytosine (HOMED6 = 0.785 [28]). The HOMED8 index is slightly larger for both **MC** (0.779) and **C** (0.791). For the neutral imino **MC**-isomers **2a**, **2b**, and **3a**-**3d**, electron delocalization slightly decreases, and the HOMED6 and HOMED8 indices (0.64-0.73) are lower than those for **1**. Additionally, favorable and unfavorable interactions between *exo* and *endo* groups affect electron delocalization. The HOMED indices are larger for isomers with favorable interactions. These effects are more important for 1-methylcytosine than for cytosine, indicating an important geometric difference between **MC** and **C**.

Positive or negative ionization decreases electron delocalization for **1**, in higher degree for its radical cation (HOMED8 = 0.599) than for its radical anion (HOMED8 = 0.700). Ionization of imino isomers induces different effects on electron delocalization. For example, the HOMED8 indices (> 0.9) for **3a**^+•^-**3d**^+•^ strongly increases, while for **2a**^+•^ and **2b**^+•^ they slightly decrease (< 0.7). For radical anions, there is no important difference in the HOMED indices between **3a**^-•^-**3d**^-•^ and **2a**^-•^-**2b**^-•^ (HOMED8 0.56-0.76). Nevertheless, different conformations of *exo* –OH group cause stronger effect on the HOMED index (ΔHOMED8 0.18) than various configurations of *exo* =NH group (ΔHOMED8 ≤ 0.04). Moreover, the charged radicals **4a** and **4b** (HOMED8 < 0.5) are less delocalized than **5a** and **5b** (HOMED8 > 0.5). Differences between the HOMED8 indices of **5a** and **4a** and also between the HOMED8 indices of **5b** and **4b** are larger for radical cations (ΔHOMED8 > 0.4) than for radical anions (ΔHOMED8 < 0.2). Due to these differences and various ionization mechanisms for individual isomers, no linear relationship exists between the geometry-based indices of positively and negatively ionized isomers of 1-methylcytosine (Fig. S1 in ESM).

**Fig. 3:**
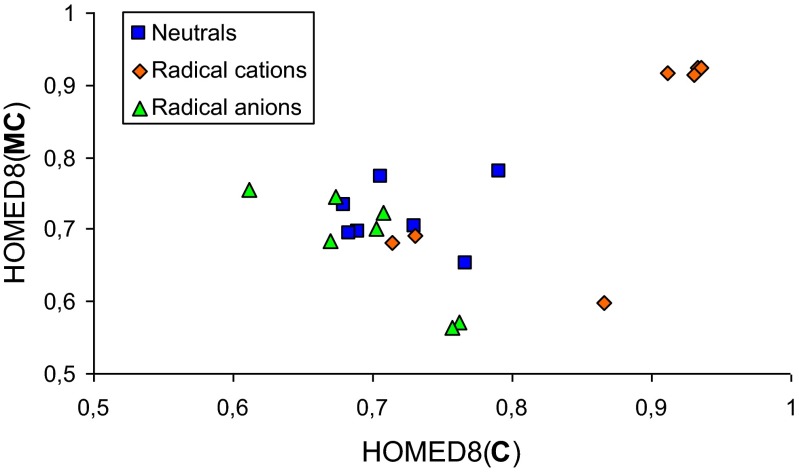
Scatter plots between the HOMED8 indices of neutral and ionized isomers of 1-methylcytosine (**MC**) and cytosine (**C**)

It should be noted here that the HOMED indices estimated for **1**-**3d** of **MC**, **MC**^+•^, and **MC**^-•^ are not parallel to those found previously for the corresponding isomers of cytosine [28]. Lack of linear relationships between the geometry-based indices (Fig. 3) indicates that the total effect of Me, substituted at N1, is completely different for individual isomers. For example, when going from cytosine to 1-methylcytosine the HOMED8 index decreases in different degree for the neutral (by 0.012), positively (0.267), and negatively ionized (0.003) amino form **1**. For the imino isomers **2a** and **2b**, the HOMED8 indices decrease for radical cations and increase for neutral and negatively charged forms. For the imino isomers **3a**-**3d**, variations of the HOMED8 indices depend on conformation of *exo* −OH group. They increase for neutral **3a** and **3c**, and they decrease for neutral **3b** and **3d**. Reverse effects occur for **3a**^-•^-**3d**^-•^. For **3a**^+•^-**3d**^+•^, which are very well delocalized for **MC**^+•^ and **C**^+•^ (HOMED8 > 0.9), variations of the HOMED8 indices are very small (< 0.02).

### Relative thermodynamic parameters

The isomers **4a**, **4b**, **5a**, and **5b** (Fig. 2) exist only for positively and negatively ionized 1-methylcytosine. For neutral **MC**, zwitterions are unstable and during optimization the initially built isomers **4a** and **4b** transform into open-ring structures with the N1−C2 bond broken, whereas **5a** and **5b** go to bicyclic structures with the additional N3−C5 bond formed. The instability of the neutral isomers **4a** and **5a** has already been signaled [93]. For all stable isomers of **MC**, neutral **1**-**3d** and ionized **1**-**5b**, real frequencies were found for minima.

Relative thermodynamic parameters {Δ(*E* + ZPE), Δ*H*, *T*Δ*S*, Δ*G*), equilibrium constants (as p*K*), and percentage contents (%) estimated at the DFT level for the **MC**, **MC**^+•^, and **MC**^-•^ isomers in *vacuo* are summarized in Table S4 (ESM). First perusal of these values clearly indicates that the canonical form **1** is the favored isomer at each oxidation state. As could be expected [28, 93, 95–99], the isomers **3a**-**3d** can be neglected in the isomeric mixture of neutral 1-methylcytosine. The **MC** mixture consists mainly of three neutral isomers: **1** (97.3 %), **2a** (2.6 %), and **2b** (0.1 %). A small amount of **2a** and **2b** has been detected in a frozen argon matrix [95]. Positive or negative ionization dramatically changes the composition of the isomeric mixture of **MC** in the gas phase.

Positive ionization of 1-methylcytosine affects the percentage contents of the three isomers: **1** (78.7 %), **2a** (18.3 %), and **2b** (3.0 %). The isomers **3a**-**3d** (< 0.001 %) are very rare forms and can be neglected in the isomeric mixture of **MC**^+•^. The isomers **4a**-**5b** (< 1⋅10^-20^%) have no importance for the structure and properties of **MC**^+•^. Since analogous calculations were performed for isolated cytosine [28], DFT results for **MC**^+•^ and **C**^+•^ can be analyzed. This analysis clearly shows that stability of **2a** strongly increases when cytosine is substituted at N1. This isomer becomes a minor one for **MC**^+•^ in the gas phase, whereas it is a rare form for **C**^+•^ (< 3 %). For negatively ionized forms, the contribution of **2a** in the isomeric mixture of **MC**^-•^ (0.6 %) is analogous to that for **C**^-•^ (0.1 %). Interestingly, the amounts of **4a**- **5b** strongly increase for **MC**^-•^, and one of them (**4a**, 1.8 %) cannot be neglected in the isomeric mixture. This mixture mainly contains the canonical tautomer **1** (97.6 %). The other imino isomers (< 0.005 %) can be neglected. A use of larger basis set (aug-cc-pVDZ) in DFT calculations for the negatively ionized isomers **1**, **2a**, and **4a** confirms the tautomeric preference (**1**, 98.2 %) for **MC**^-•^.

**Fig. 4:**
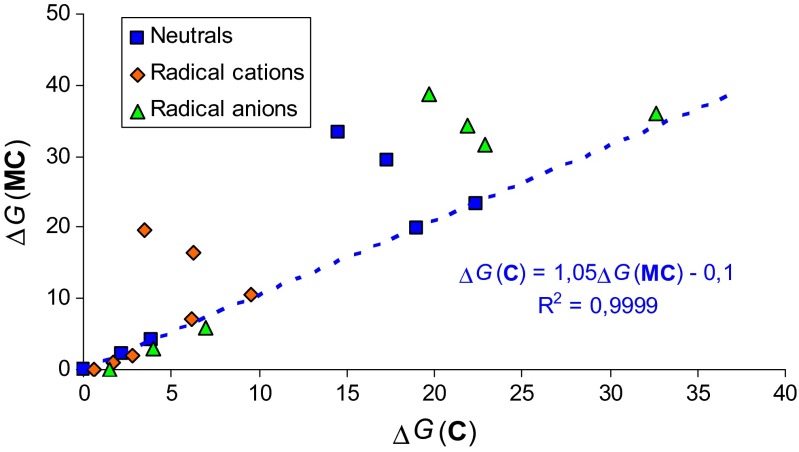
Plots of the gas-phase relative Gibbs energies (Δ*G* in kcal mol^-1^) of neutral and ionized forms of 1-methylcytosine (**MC**) against those of cytosine (**C**)

Relative thermodynamic parameters for the **MC**, **MC**^+•^, and **MC**^-•^ isomers can be plotted against those previously calculated at the same level of theory for the corresponding isomers of cytosine [28]. The plots of the calculated Δ*G* values for **MC**, **MC**^+•^, and **MC**^-•^ isomers against those for cytosine (Fig. 4) show significant differences for **3b** and **3d**, for which the *exo* −OH group unfavorably interacts with Me at N1 in **MC**, and favorably interacts with N1 in **C**. These opposite intramolecular interactions lead to strong deviations of points referring to these two isomers. For other **MC** isomers, the Δ*G* values correlate quite well with those of the corresponding **C** isomers. For example, the slope of linear relationship for neutral isomers (1.05) and the correlation coefficient (r = 0.999_9_) are close to unity. The same is true for ionized isomers, radical cations and radical anions. Generally, relative parameters for neutral and ionized isomers of 1-methylcytosine (except **3b** and **3d**) are parallel to those of cytosine.

**Fig. 5:**
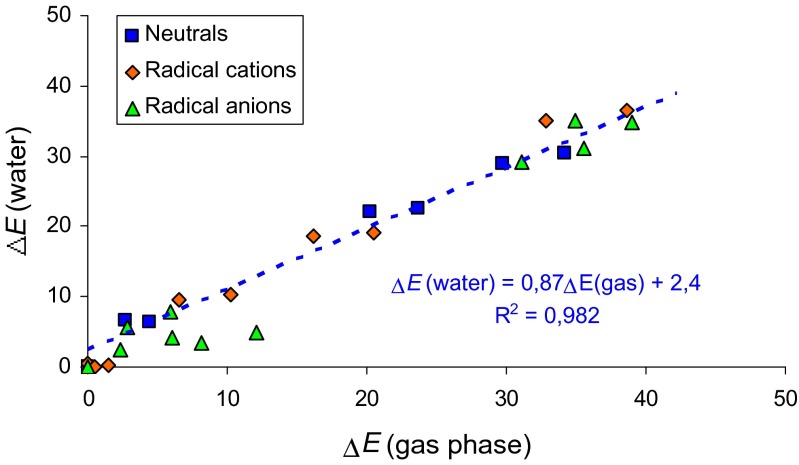
Plot of the relative energies (Δ*E* including ΔZPE, in kcal mol^-1^) of neutral and ionized forms of 1-methylcytosine calculated in aqueous solution against those found in the gas phase

An application of the PCM(water) model to the DFT-optimized geometries of **MC** isomers displays an interesting effect of medium polarity (Table S5 in ESM, ZPEs estimated at the DFT level were applied to the total electronic energies calculated at the PCM(water) level). For neutral **MC** isomers, the canonical form **1** is favored in aqueous solution (100 %, assuming that thermal corrections and entropy terms in aqueous solution are the same as those in the gas phase). Relative energies of **2a** and **2b** are larger than 6 kcal mol^-1^, and thus their contributions in the isomeric mixture of neutral **MC** may be lower than 0.01 %. Relative energies of **3a**-**3d** do not change very much when going from nonpolar (gas phase) to polar environment (aqueous solution). Their amounts can be neglected in the isomeric mixture of **MC**. Generally, the relative energies calculated at the PCM(water) level are almost parallel to those estimated at the DFT level, and a good linear relationship is found (r = 0.991) for neutral isomers of **MC** (Fig. 5). The slope of this line (0.87) is slightly lower than unity indicating some attenuation of isomerization effects in aqueous solution.

For **MC**^+•^ isomers, polar solvent seems to influence tautomeric equilibria and isomeric preferences. In aqueous solution, the rare isomer **2a** seems to be the favored one (45.0 %). Its rotamer **2b** also contributes in the isomeric mixture (32.0 %). The canonical form **1** is rather a minor form (22.9 %). The other iminol isomers can be neglected. A change of the relative energies for **MC**^+•^ isomers when going from one extreme environment to the other one slightly destroys the linear relation observed for neutral isomers (Fig. 5). Larger deviations of points are found for **MC**^-•^ isomers. The canonical form **1** predominates for **MC**^-•^ (97.9 %) in aqueous solution. The contribution of the rare isomers **2a** and **2b** is very small (< 0.01 %). The very rare isomers **3a**-**3d** (<< 0.01 %) can be neglected. The isomer **4a** (1.7 %) significantly contributes in the isomeric mixture of **MC**^-•^. The amounts of **4b** (0.3 %), **5a** (0.08 %), and **5b** (0.03 %) seem to be larger than those in the gas phase but they do not exceed 0.5 %. Generally, polarity of water affects the relative energies of imino isomers. It also reduces differences between the relative energies for the rotamers **a** and **b** of **4** and **5** from *ca.* 6 kcal mol^-1^ in *vacuo* to *ca.* 1 kcal mol^-1^ in aqueous solution.

### Properties of the favored neutral and ionized isomeric mixtures

**Scheme 1:**
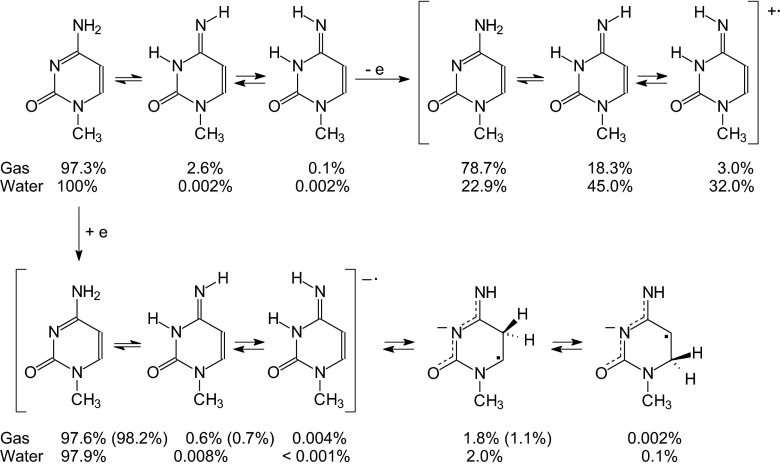
Composition of the isomeric mixture for neutral and ionized 1-methylcytosine estimated at the B3LYP/6-311+G(d,p) level (data for selected radical anions found at the B3LYP/aug-cc-pVDZ level are given in parentheses)

Considering only major, minor, and rare isomers (> 0.001 %) for neutral and ionized 1-methylocytosine, the following ionization processes can be drawn (Scheme 1). In *vacuo*, the neutral and positively ionized isomeric mixtures of **MC** contain three isomers (**1**, **2a**, and **2b**), whereas at least five isomers (**1**, **2a**, **2b**, **4a**, and **5a**) can be considered for the negatively ionized molecule. For each oxidation state, the canonical isomer **1** is the favored form for 1-methylcytosine. The rare isomer **2a** seems to be present in the highest amount (*ca*. 20 %) only for **MC**^+•^. For **MC** and **MC**^-•^, its contribution in the isomeric mixture is considerably lower (< 3 % and < 1 %, respectively). In aqueous solution, composition of the isomeric mixture dramatically changes only for **MC**^+•^. In this case, the rare isomer **2a** becomes the favored one (45 %), and the canonical isomer **1** is rather a minor form (23 %). **2b** also has an important contribution (32 %). For neutral and negatively ionized 1-methylcytosine, the isomer **2a** is indeed the rare form (< 1 %). The isomers **4a**-**5b**, strongly stabilized by an unpaired electron, can also be considered as rare forms for **MC**^-•^ (≤ 2 %).

The positive and negative ionization processes can be characterized by the ionization potential (IP) and by the electron affinity (EA), respectively. Unfortunately, there are no experimental data for 1-methylcytosine. However, there are experimental data for canonical cytosine (IP = 8.45 eV [111] and EA = 0.23 eV [112]) and for unsubstituted pyrimidine (IP = 9.33 eV [3] and EA < −0.25 eV [113]). It should be noted that the level of theory applied here {(B3LYP/6-311+G(d,p)} reproduce well the IP (9.18 eV) and EA (−0.14 eV) for pyrimidine. Moreover, the DFT-calculated EA for pyrimidine is close to that (−0.17 eV) found at the G3MP2B3 level [3]. This confirms that the level of theory applied here is sufficient and appropriate for neutral and ionized species, radical cations, and radical anions.

If we assume that removing or adding one electron does not very much affect the structure of **MC** isomers, we can calculate in *vacuo* and in aqueous solution the microscopic IP and EA for the individual tautomers-rotamers **1**-**3d**, taking the energies of neutral and ionized forms into account (Table S6 in ESM). The macroscopic parameters referring to the isomeric mixture of **MC** (Scheme 1) can also be estimated. They are as follows: IP = 8.2 and 6.3 eV and EA = −0.4 and 1.8 eV in the gas phase and aqueous solution, respectively. Polarity of water changes the ionization parameters (IP and EA) by *ca*. 2 eV. An analogous hydration effect has been found for nucleic acid bases and their models [16–19, 24, 26]. The negative adiabatic EA value for **MC**, close to that for pyrimidine [113], confirms our spin-density analysis (Table S2 in ESM). Upon negative ionization the pyrimidine ring preferentially takes one electron. Additionally, the negative EAs for **MC** isomers indicate that negatively ionized forms, possessing energies very close to that of neutral ones, do not exist for a suitable time period. EA measurements cannot be performed with good precision. There are also other molecules which possess negative EAs in the gas phase [3, 15, 113]. Nevertheless, they can be investigated in solution or clusters, for which the EAs are positive.

### Basicity and acidity of neutral and ionized MC in the gas phase

There are no solvent molecules in the gas phase and acid-base properties of organic compounds depend only on functional groups. Acidity or basicity can be described by thermodynamic parameters of the corresponding deprotonation or protonation reaction, Δ*H* and Δ*G*, which differ by the entropy term, Δ*G* = Δ*H* - *T*Δ*S* [3, 114]. For deprotonation of AH group (AH → A^-^ + H^+^), these thermodynamic parameters refer to the deprotonation enthalpy (DPE = Δ*H*_acid_) and to the gas-phase acidity (GA = Δ*G*_acid_). For protonation of B group (B + H^+^ → BH^+^), the proton affinity (PA = − Δ*H*_base_) and the gas-phase basicity (GB = − Δ*G*_base_) were proposed. The use of different symbols for acids and bases is only formal, because DPE(AH) = PA(A^-^), GA(AH) = GB(A^-^), PA(B) = DPE(BH^+^), and GB(B) = GA(BH^+^). Both, the DPE and PA values are on the same Δ*H* scale, and the GA and GB values are on the same Δ*G* scale. Stronger acid has lower DPE and GA values and stronger base has larger PA and GB values.

In the case of neutral 1-methylcytosine, experimental gas-phase acidity and gas-phase basicity parameters have been determined in 2008 by Lee and co-workers [97]. The authors used a Fourier transform ion cyclotron resonance mass spectrometer and the bracketing method, and derived the following macroscopic acid-base parameters: DPE = 349 ± 3 and GA = 342 ± 3 kcal mol^-1^ for deprotonation of neutral **MC** to its monoanion **MC-H**^**+**^ (**MC** → **MC-H**^**+**^ + H^+^), and PA = 230 ± 3 and GB = 223 ± 3 kcal mol^-1^ for protonation of neutral **MC** to its monocation **MCH**^**+**^ (**MC** + H^+^ → **MCH**^+^). Comparison of these experimental data with those found for other organic compounds in the gas phase [3] shows that 1-methylcytosine displays acidity close to that of pyrrole (DPE = 359.6 and GA = 351.8 kcal mol^-1^) and basicity close to that of imidazole (PA = 225.3 and GB = 217.3 kcal mol^-1^) and 2-aminopyridine (PA = 226.4 and GB = 218.8 kcal mol^-1^). Both pyrrole and 1-methylcytosine belong to the family of NH acids, for which the NH group is deprotonated. On the other hand, imidazole, 2-aminopyridine, and 1-methylcytosine belong to the family of N bases which contain the amidine group >N−C=N− with the imino N site preferentially protonated.

**Scheme 2:**
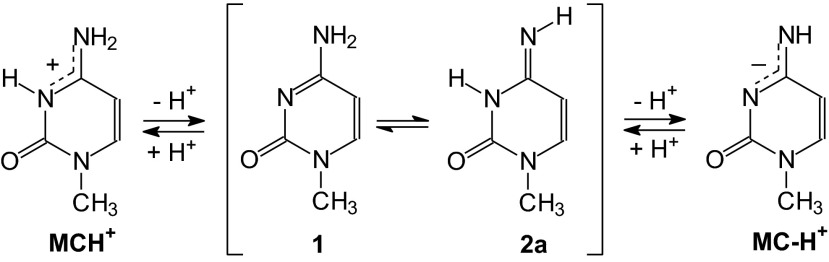
Protonation/deprotonation of the neutral canonical (**1**) and rare (**2a**) isomers of 1-methylcytosine to the corresponding monocation (**MCH** ^**+**^) and monoanion (**MC-H** ^**+**^)

Using the B3LYP functional and the 6-31+G(d) basis set, Lee and co-workers [97] additionally found the following microscopic parameters for deprotonation of the neutral canonical form **1** at N8 and for its protonation at N3: DPE = 348.3, GA = 340.3, PA = 230.0, and GB = 222.4 kcal mol^-1^, respectively. For the neutral rare form **2a**, these parameters for deprotonation at N3 and for protonation at N8 are as follows: DPE = 350.7, GA = 344.5, PA = 232.9, and GB = 224.9 kcal mol^-1^, respectively. Deprotonation of **1** and **2a** leads to the same monoanion **MC-H**^**+**^, and their protonation goes to the same monocation **MCH**^**+**^ (Scheme 2). For comparison, at the B3LYP/6-311+G(d,p) level we found the following values for **1** (355.8, 347.0, 232.0, and 224.2 kcal mol^-1^) and **2a** (353.2, 344.9, 234.6, and 226.3 kcal mol^-1^).

**Scheme 3:**
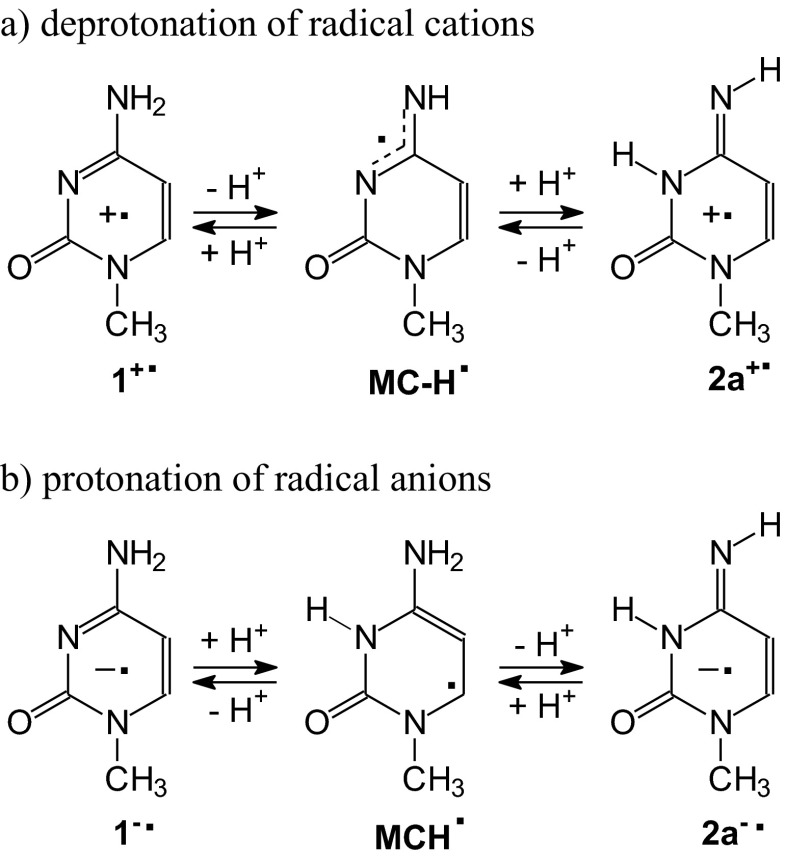
Deprotonation of radical cations (**a**) and protonation of radical anions (**b**) to the corresponding neutral radicals of 1-methylcytosine

To our knowledge, gas-phase acidity and gas-phase basicity for radical cations and radical anions of 1-methylcytosine have not yet been reported in the literature. Nevertheless, they can be estimated on the basis of our DFT calculations performed for selected neutral and charged radicals. According to Sevilla and co-workers experiments performed for nucleobases and their models [32, 107], deprotonation of radical cation of 1-methylcytosine and protonation of its radical anion can lead to the corresponding neutral radical: **MC**^+•^ − H^+^ → **MC-H**^•^ and **MC**^-•^ + H^+^ → **MCH**^•^. Acidity and basicity parameters for these deprotonation and protonation reactions in the gas phase can be predicted from the calculated thermodynamic parameters for the corresponding radicals by the same procedure applied for neutral 1-methylcytosine (see Computational details in ESM). For the canonical and rare isomers of 1-methylcytosine, their radical cations **1**^+•^ and **2a**^+•^ can be deprotonated to the same neutral radical **MC-H**^•^, whereas their radical anions **1**^-•^ and **2a**^-•^ can be protonated to the other neutral radical **MCH**^•^ (Scheme 3). At the DFT level, the following microscopic parameters were estimated in the gas phase for these reactions (in kcal mol^-1^): DPE(**1**^+•^) = 230.6, GA(**1**^+•^) = 223.4, DPE(**2a**^+•^) = 230.4, GA(**2a**^+•^) = 222.5, PA(**1**^-•^) = 350.8, GB(**1**^-•^) = 343.2, PA(**2a**^-•^) = 353.5, and GB(**2a**^-•^) = 346.2, respectively.

**Fig. 6:**
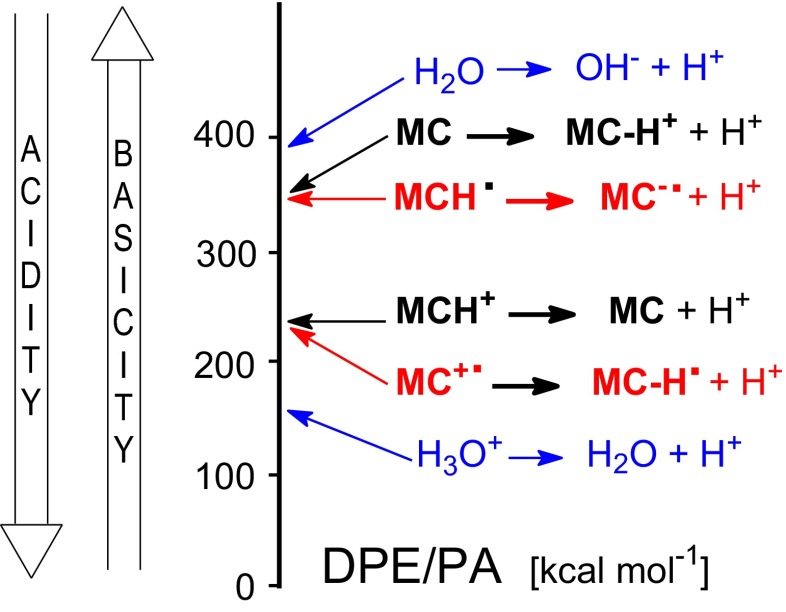
Comparison of the DFT-estimated proton affinities and deprotonation enthalpies for 1-methylcytosine and its radicals with experimental data for water

Interestingly, gas-phase acidities of the radical cations **1**^+•^ and **2a**^+•^ are close to that of the monocation **MCH**^**+**^, and gas-phase basicities of the radical anions **1**^-•^ and **2a**^-•^ are close to that of the monoanion **MC-H**^**+**^. In other words, gas-phase basicity of the neutral radical **MC-H**^•^ and gas-phase acidity of the neutral radical **MCH**^•^ are close to those of neutral 1-methylcytosine (Fig. 6). Moreover, the radical cations **1**^+•^ and **2a**^+•^ are stronger bases than neutral water (PA = 165.2 and GB = 157.7 kcal mol^-1^ [3]), indicating that water cannot deprotonate them in the gas phase (apolar environment). Water is too weak a base. Water is also too weak an acid (DPE = 390.3 and GA = 383.7 kcal mol^-1^ [3]), and cannot protonate the radical anions **1**^-•^ and **2a**^-•^. Other compounds, being stronger bases and stronger acids than 1-methylcytosine in the gas phase, can be considered in the future for deprotonation of its radical cations and for protonation of its radical anions in an apolar environment.

**Fig. 7:**
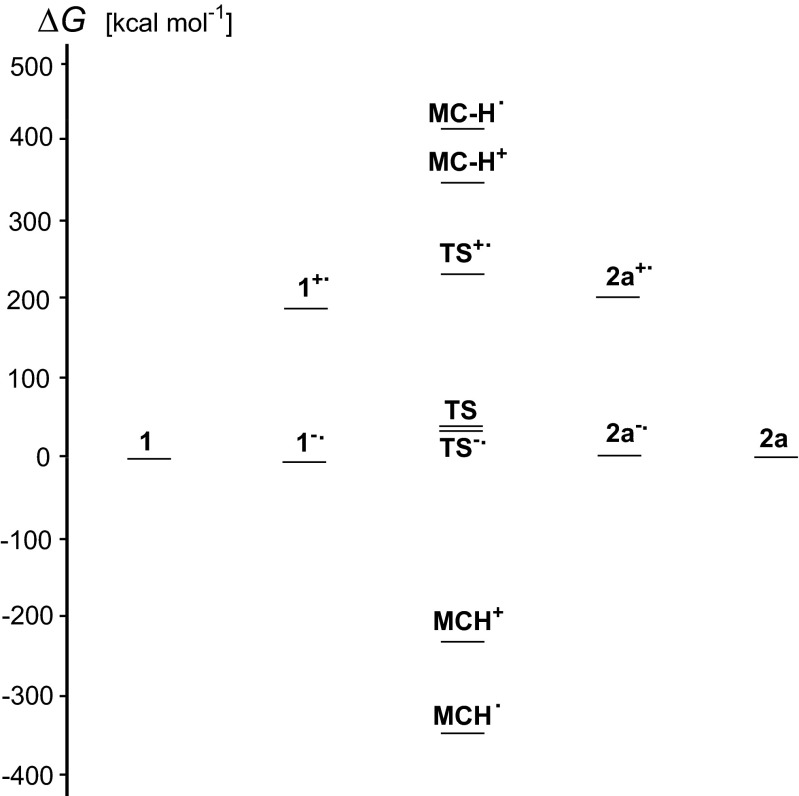
Variations of the DFT-calculated relative Gibbs energies (in kcal mol^-1^) of various **MC** structures when going from the amino (**1**) to imino (**2a**) isomer

### How the “canonical” form of MC can be converted into its “rare” form?

**Scheme 4:**
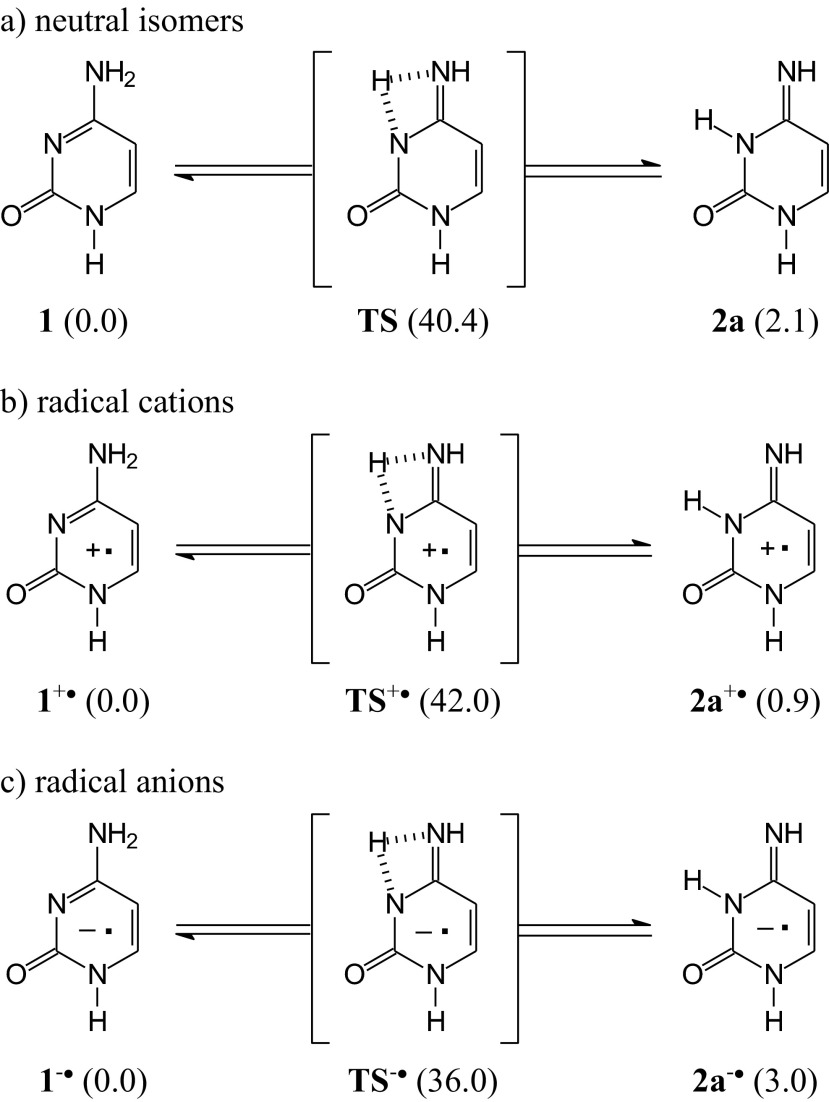
One-step intramolecular proton-transfer considered in the gas phase between selected neutral (**a**), positively (**b**), and negatively ionized (**c**) isomers of 1-methylcytosine (relative Gibbs energies calculated at the DFT level are given in kcal mol^-1^)

Various routes can be considered for transformation of the canonical tautomer **1** into the rare isomer **2a** for neutral and ionized 1-methylcytosine, one-step, two-steps, or multi-steps amino-imino conversion. In the gas phase, the labile proton can be transferred intramolecularly from N8 to N3 through the corresponding transition state (Scheme 4). We found three transition states between the neutral isomers **1** and **2a** (**TS**) and their positively (**TS**^+•^) and negatively (**TS**^-•^) ionized forms at the DFT(B3LYP)/6-311+G(d,p) level. Each of these transition states possesses one imaginary frequency. Inclusion of thermal corrections when proceeding from 0 to 298 K and entropy terms, leads to the following relative Gibbs energies (relative to the canonical tautomer) for **TS**, **TS**^+•^, and **TS**^-•^: Δ*G* = 40.4, 42.0, and 36.0 kcal mol^-1^, respectively. The estimations indicate that positive ionization slightly increases the energetic-barrier for tautomeric conversion between the amino and imino isomers whereas negative ionization slightly decreases it. Our DFT calculations show additionally that the energetic-barrier for the neutral tautomers **1** and **2a** is slightly lower than that found by Fogarasi [76] (Δ*E* = 45-50 kcal mol^-1^ at the B3LYP, MP2, and various CC levels) for tautomeric conversion in the parent system, formamidine (HN=CH−NH_2_/H_2_N−CH=NH). When one water molecule participates in this conversion, the energetic-barrier diminishes by 25-30 kcal mol^-1^. An analogous decrease of the energetic-barrier for amino-imino conversion may be expected for the **MC** isomers.

Tautomeric amino-imino conversion between the canonical and rare tautomers of 1-methylcytosine can also run through two-steps proton-transfer reaction. For neutral forms, the monoanion **MC-H**^**+**^ or monocation **MCH**^**+**^ can be an intermediate product between the isomers **1** and **2a** as shown in Scheme 2. For radical ions, tautomeric conversion between **1**^+•^ and **2a**^+•^ can run through the neutral radicals **MC-H**^•^ and that between **1**^-•^ and **2a**^-•^ can run through the other neutral radical **MCH**^•^ as shown in Scheme 3.

**Scheme 5:**
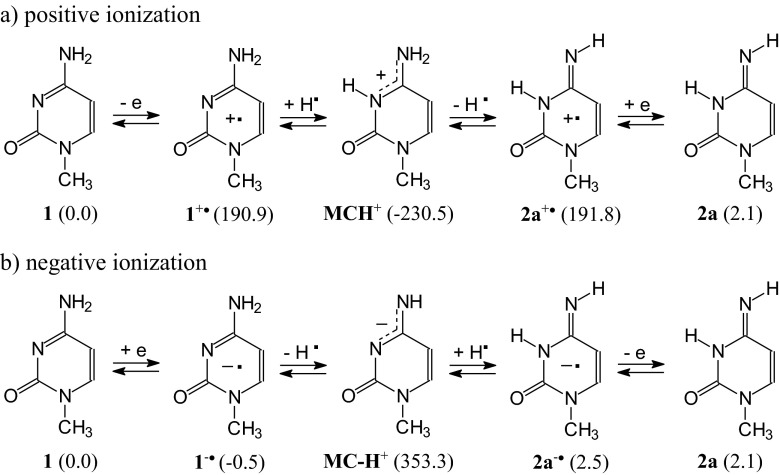
Multi-steps conversion considered in the gas phase between isomers of 1-methylcytosine *via* the protonated **MC-H** ^+^ (**a**) and deprotonated **MC-H** ^+^ (**b**) forms (relative Gibbs energies calculated at the DFT level are given in kcal mol^-1^)

Investigating gas-phase basicities of organic compounds by quantum-chemical methods, Makisć and co-workers [72] analyzed the following steps from neutral (B) to monoprotonated (BH^+^) forms: positive ionization of base (B – e → B^+•^), formation of hydrogen atom (H^+^ + e → H^•^), and formation of conjugate acid (B^+•^ + H^•^ → BH^+^). These steps are in agreement with experiment. Gas-phase basicities can be measured using various MS techniques with positive ionization, and ions B^+•^ and BH^+^ detected and analyzed [3, 57, 114]. Taking into account the MS experiments and the “triadic analysis”, tautomeric conversion between **1** and **2a** can also run through **MCH**^+^, **1**^+•^, and **2a**^+•^. An analogous tautomeric conversion between **1** and **2a** can be proposed for negative ionization in the gas phase, where **1**^-•^, **2a**^-•^ and **MC-H**^+^ can be intermediates (Scheme 5).

**Scheme 6:**
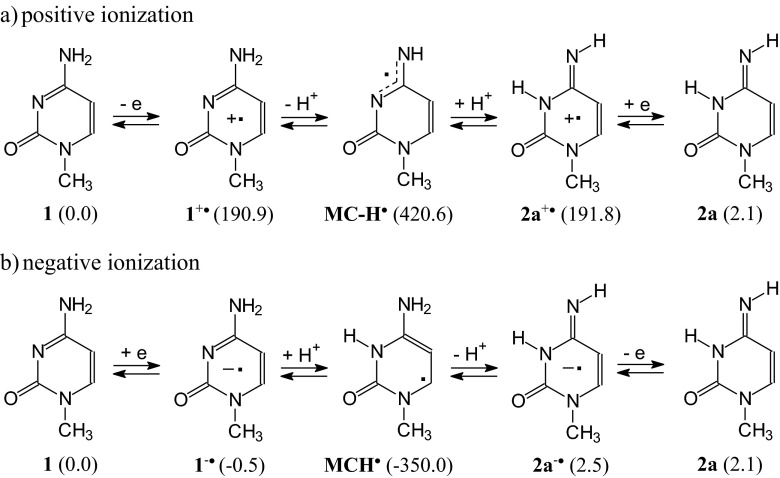
Multi-steps conversion considered in the gas phase between isomers of 1-methylcytosine *via* the neutral radicals **MC-H** ^•^ (**a**) and **MCH** ^•^ (**b**) (relative Gibbs energies calculated at the DFT level are given in kcal mol^-1^)

Another conversion-route between the ionized canonical and rare isomer of 1-metylcytosine can be proposed on the basis of Choi et al. experiments for guanidine radical cations [12]. The authors observed the neutral radical as an intermediate between two ionized isomers. On the other hand, Sevilla, Tureček, and their co-workers [5, 6, 32, 56, 106, 107] proposed a deprotonation reaction for radical cations and protonation reaction for radical anions, both leading to the corresponding neutral radicals. Taking these experimental observations into account, tautomeric conversion between the ionized canonical and rare isomers of 1-methylcytosine can also run through **MC-H**^•^ or **MCH**^•^ (Scheme 6).

The route of proton-transfer between the canonical and rare isomers of 1-methylcytosine may depend on the method of investigation. For example, in matrix isolation IR, MW, and REMPI experiments, the intramolecular proton-transfer proposed in Scheme 4a may take place for the neutral isomers. During experimental MS gas-phase acidity/basicity determinations, tautomeric conversion may run through the monocation **MCH**^**+**^ or monoanion **MC-H**^**+**^ (Scheme 5). The neutral radicals **MC-H**^•^ or **MCH**^•^ may be intermediates between the radical ions (Scheme 6) in zero kinetic energy photoelectron spectroscopic studies.

## Conclusions

Our calculations carried out in the gas phase at the DFT level and in aqueous solution at the PCM(water) level for the isomeric mixture of neutral and ionized 1-methylcytosine show clearly that positive and negative ionization change both the geometry- and energy-based parameters. Electron delocalization in the pyrimidine ring and also in the whole tautomeric system dictates the isomeric preference only for neutral **MC**. The most delocalized amino isomer **1** (HOMED8 = 0.779) is favored for **MC**. When going from the neutral to charged radicals, electron delocalization changes dramatically. Due to completely different Me effects for individual **MC** isomers, the changes are not parallel to those observed earlier for cytosine (Fig. 3) [28]. Only relative thermodynamic parameters for **MC** isomers correlate well with those for the corresponding isomers of cytosine (Fig. 4). Some exceptions are the isomers **3b** and **3d** with unfavorable interactions between OH and Me groups.

In the gas phase, which models apolar environments (lipids), positive or negative ionization does not change the tautomeric preference (Scheme 1). Ionization influences the isomeric mixture. The canonical isomer **1** is favored for **MC**, **MC**^+•^, and **MC**^-•^. The rare isomer **2a**, responsible for DNA mutation [31], appears in detectable amounts in the isomeric mixture of **MC**, **MC**^+•^, and **MC**^-•^ with the highest contribution for **MC**^+•^. Its percentage content increases in aqueous solution, which models a more polar environment (enzymes, receptors, proteins, nucleoproteins, *etc*.). The rare isomer **2a** becomes the favored form for **MC**^+•^, while the canonical tautomer **1** is only a minor form. For **MC**^-•^, the percentage contents of the radical anions **4a**-**5b** are larger than that of **2a**. They are well stabilized by an unpaired electron. Polarity of water does not affect their contributions in the isomeric mixture. Positive ionization strongly increases the energies of **4a**-**5b** such that they can be neglected in the isomeric mixture of **MC**^+•^. For neutral **MC**, they do not exist. Four rotamers of **3** can be neglected at each oxidation state in both extreme environments.

The most important effects observed for tautomeric 1-methylcytosine seem to result from interactions of **MC** with positively ionized agents. They are completely different from those observed previously for isolated cytosine and better describe some tendencies of cytosine included in DNA to intramolecular proton-transfer than isolated cytosine. Interestingly, gas-phase acidities of radical cations of **MC** are close to that of its monocation, and gas-phase basicities of radical anions are close to that of its monoanion. Water in the gas phase is too weak a base to deprotonate **MC**^+•^ and also it is too weak an acid to protonate **MC**^-•^. This observation suggests that radical ions may live a longer time in an apolar than polar environment and may be responsible for changes in nucleic acids. Figure 7 summarizes the DFT-calculated relative Gibbs energies of possible structures which 1-methylcytosine may adopt when proceeding from the canonical to rare isomer.

## Electronic supplementary material

Below is the link to the electronic supplementary material.

ESM 1 (DOC 646 kb)
