# Supplementary material for: Effects of ionization on stability of 1-methylcytosine — DFT and PCM studies
Source: J Mol Model. 2016 Jun 3;22:146. doi: 10.1007/s00894-016-3020-2 (PMC4893064; doi:10.1007/s00894-016-3020-2)
Supplement: Supplementary file 1 — (DOC 646 kb) [file 894_2016_3020_MOESM1_ESM.doc]

Electronic Supplementary Material

**Effects of positive and negative ionizations on stability
of 1-methylcytosine – DFT and PCM studies**

**Ewa D. Raczyńska,^a^* Piotr Michalec,^b^ Marcin Zalewski,^b^ Mariusz Sapuła^c^**

*^a^Department of Chemistry,Warsaw University of Life Sciences (SGGW), 02-776 Warszawa, Poland*

*^b^Faculty of Agriculture and Biology, SGGW, 02-776 Warszawa, Poland*

*^c^Interdisciplinay Department of Biotechnology, SGGW, 02-776 Warszawa, Poland*

*E-mail: ewa_raczynska@sggw.pl, Tel.: +48 225937623, Fax: +48 225937635*

**Choice of methods**

Two different types of intramolecular proton-transfers (IPTs) from acidic to basic sites are possible for polyfunctional biomolecules: IPTs leading to isomers named “zwitterions” when the acidic and basic sites are not conjugated, and IPTs going to isomers called “prototropic tautomers” when the sites are conjugated. Tautomers differ by positions of labile proton(s) and bond(s), and can be represented by hybrids of various resonance structures. Zwitterions differ only by positions of labile proton(s). In the literature, IPTs are frequently described by thermodynamic parameters such as relative energies (Δ*E* at 0 K), relative enthalpies (Δ*H* at 298.15 K), relative Gibbs energies (Δ*G* = Δ*H* − *T*Δ*S* at 298.15 K), and/or equilibrium constants (as p*K* = Δ*G*/2.303R*T*). Equilibrium constants between individual isomers (*e.g*., *K* = x/y between two isomers for which their amounts are equal to x and y), determine, by definition, their percentage contents {*x* = *K*/(1 + *K*) and y =1/(1 + *K*)} in their isomeric mixture.

To study the structural and energetic parameters of all possible individual tautomers and zwitterions, quantum-chemical methods can be applied, because experimental techniques (UV, IR, Raman, NMR, MW, MS, *etc*.) cannot give complete information on all possible isomers and on all possible isomeric equilibria. Among various quantum-chemical methods, the density functional theory (DFT) method [65] with the three-parameter hybrid functional of Becke [66] and the non-local correction functional of Lee, Yang, and Parr (B3LYP) [67] have been the most frequently used for proton-transfer reactions [22, 72, 73]. The B3LYP functional is sufficient for geometry optimization as well as for estimation of relative energetic parameters. This method has been successfully employed for various aliphatic and aromatic systems for which keto-enol, imine-enamine, imine-amine, and amide-iminol conversions occur in the gas phase [22]. It has been applied for neutral parent systems such as acetaldehyde-vinyl alcohol, acetaldimine-vinylamine, formamide-formamidic acid, and formamidine [74-76] as well as for more complex tautomeric aromatic systems such as neutral and redox phenol, aniline, their aza derivatives, nucleobases and their model compounds [21, 24, 26-28, 56, 77-81, 107]. For example, the DFT-calculated relative energies for acetaldehyde-vinyl alcohol, acetaldimine-vinylamine, and formamide-formamidic acid are close to those calculated at the MP2, G2, CCSD, CCSD(T), and CCSDT levels [75, 76]. Differences are not larger than 1 kcal mol^-1^. In the case of aniline, the DFT-calculated relative Gibbs energies for enamine-imine conversions differ from the G2 ones by 2-3 kcal mol^-1^ [79]. A use of different basis set with various polarization and diffuse functions {6-311+G(d,p), 6-311++G(3df,3pd), and aug-cc-pVDZ} has no important effect on the B3LYP-calculated relative thermodynamic parameters for aniline. For five-membered imidazole (building block of purine, adenine, and guanine), differences between the DFT-, G2-, and G3B3-calculated relative Gibbs energies for enamine-imine conversions are not larger than 2 kcal mol^-1^ [82].

Schaefer and co-workers [15], considering various quantum-chemical methods for investigations of radical ions and for calculations of electron affinities (EAs), recommended the B3LYP functional. An application of this functional leads to the lowest differences between the computed and experimental EA values [15]. Sevilla and co-workers also used the B3LYP functional for investigation of one-electron loss and one-electron gain in nucleobases and their pairs [18, 52, 53, 56, 107]. Taking into account the literature data for neutral and charged forms of nucleobases and for their model compounds and also recommendation of Schaefer [15] and application of Sevilla [18, 52, 53, 56, 107], we decide to apply the B3LYP functional [65-67] and the 6-311+G(d,p) basis set [68]. We confirmed by computations that this level of theory reproduces well the IP and EA values for pyrimidine – the building block of 1-methylcytosine. For selected radical anions, the larger aug-cc-pVDZ basis set [71] was also applied. For aromatic systems, thermal corrections and entropy therms are very close for individual isomers and relative energies, relative enthalpies, and relative Gibbs energies are not very different, *i.e*., ΔE ≈ Δ*H* = Δ*G* [22, 24-28, 77-82]. To study the solvent effects, the polarizable continuum model (PCM) [69, 70] was employed to the neutral and charged forms optimized at the B3LYP/6-311+G(d,p) level. The PCM method has already been tested for tautomeric systems and for charged radicals including nucleobases [19, 24, 26, 28, 56, 83-85]. Since it does not take into account specific interactions, it was not applied for deprotonation of radical cations and for protonation of radical anions occurring at particular pH [32].

To properly determine the distribution of n- and π-electrons for isomers of 1-methylcytosine, the geometry-based HOMED (harmonic oscillator model of electron delocalization) procedure [87, 88] was applied to the geometries optimized at the DFT(B3LYP)/6-311+G(d,p) level. The HOMED index was chosen, because it describes well any type of resonance conjugation, weak σ-π hyperconjugation, medium n-π and π-π conjugation, and also aromaticity [88]. From mathematical point of view, the HOMED procedure is analogous to that of the original HOMA index, proposed in 1972 by Kruszewski and Krygowski to describe quantitatively π-electron delocalization for homo- and heteroaromatic systems [89, 90]. One difference is its parametrization [88]. An abbreviation HOMED was proposed in 2006 for the modified index [87], but it may be also abbreviated as moHOMA (modified original HOMA) or simply HOMA.

The HOMA index, reformulated by Krygowski in 1993 (rHOMA) [91], seems to be inappropriate for π-electron compounds containing heteroatoms, because different measures of π-electron delocalization were employed for reference CC, CX, and XY bonds [88, 91]. The rHOMA can only be used for systems containing the same type of bonds, *e.g*., hydrocarbons [88]. It should be mentioned here that the HOMHED (harmonic oscillator model for heterocyclic electron delocalization) index, proposed in 2012 by Frizzo and Martins [92], is based on hypotheses and ideas of the HOMED index [87, 88]. One difference is an application of statistical reference CC, CX, and XY bond lengths in the HOMHED procedure. The use of statistical reference bond lengths leads to some kind of statistical HOMHED values which do not describe well the real electron delocalization in heteroatomic systems. For this reason, the HOMHED index was not applied here.

**Computational details**

Geometries of all neutral (**MC**), protonated (**MCH**^+^), and deprotonated (**MC-H**^+^) forms of 1-methylcytosine, its neutral (**MC-H**^•^ and **MCH**^•^) and charged radicals (**MC**^+•^ and **MC**^-•^), and also transition states (T**S**, **TS**^+•^, and **TS**^-•^) for selected conversions were fully optimized in the gas phase without symmetry constraints at the DFT(B3LYP)/6-311+G(d,p) level [65-68]. Charged radicals were theoretically derived by removing from and adding to neutral isomers one electron. These transformations refer to positive and negative ionization, respectively. The restricted B3LYP functional was used for neutral, protonated and deprotonated forms, and the unrestricted B3LYP functional was applied for radicals. For all stable structures optimized in their ground states, frequencies were calculated, first to prove that the structures are minima, and next to estimate the corresponding zero point energies. For selected radical anions, calculations were also performed using the aug-cc-pVDZ basic set [71]. Calculations in aqueous solution were carried out without symmetry constraints at the PCM(water)//DFT(B3LYP)/6-311+G(d,p) level [69, 70].

Thermodynamic parameters {ΔE, Δ*H*, Δ*G*, and p*K*} were calculated using the same level of theory as that applied for geometry optimization. The Δ*G*s include changes in the electronic energy, zero-point energy (ZPE), and thermal corrections to the energy and entropy (vibrational, rotational, and translational). The theoretical adiabatic ionization potential {IP = *E*(optimized radical cation) - *E*(optimized neutral)} and the theoretical adiabatic electron affinity {EA = *E*(optimized neutral) - *E*(optimized radical anion)} were calculated for individual tautomers-isomers and also for the mixture of major isomers, taking the total energies of neutral and charged forms at their respective equilibrium nuclear configurations. Proton affinities {PA = *H*(optimized neutral or radical anion) + *H*(H^+^) – *H*(optimized protonated form or neutral radical)}, gas-phase basicities {GB = *G*(optimized neutral or radical anion) + *G*(H^+^) – *G*(optimized protonated form or neutral radical)}, deprotonation enthalpies {DPE = *H*(optimized deprotonated form or neutral radical) + *H*(H^+^) – *H*(optimized neutral or radical cation)}, and gas-phase acidities {GA = *G*(optimized deprotonated form or neutral radical) + *G*(H^+^) – *G*(optimized neutral or radical cation)} were also estimated for selected neutral **MC** isomers and for its radicals using *H*(H^+^) = 5/2*RT* = 1.5 kcal mol^-1^ and *G*(H^+^) = *H*(H^+^) – *TS*(H^+^) = 6.3 kcal mol^-1^ [3, 114]. All calculations were performed using the Gaussian 03 program [86].

Since the same resonance phenomenon takes place for neutral molecules, ions, and radicals, the HOMED indices were estimated for neutral and ionized forms of **MC** using the following equation [88]: HOMED = 1 - {α(CC)⋅Σ[*R*_o_(CC) - *R*_i_(CC)]^2^ + α(CX)⋅Σ[*R*_o_(CX) - *R*_i_(CX)]^2^}/*n.* In this equation, α are normalization constants, *R*_o_ are the optimum bond lengths (assumed to be realized for fully delocalized systems), *R*_i_ are the running bond lengths, and *n* is the number of bonds taken into account. For **MC** isomers, six bonds for the pyrimidine ring, and eight bonds for the whole tautomeric system, including *exo* –OH/=O and –NH_2_/=NH groups, were taken into account. The normalization α constants for the even number of bonds were calculated from the following equation [88, 91]: α = 2 ⋅ [(*R*_o_ - *R*_s_)^2^ + (*R*_o_ - *R*_d_)^2^]^-1^, where *R*_s_ and *R*_d_ are the reference single and double bonds, respectively. The following *R*_s_, *R*_d_, and *R*_o_ values (in Å), calculated at the B3LYP/6-311+G(d,p) level for reference molecules, were taken here [88]: 1.530 (ethane), 1.329 (ethene) and 1.394 (benzene) for the CC bonds, 1.466 (methylamine), 1.267 (methylimine) and 1.334 (1,3,5-triazine) for CN bonds, and 1.424 (methanol), 1.202 (formaldehyde) and 1.281 (protonated carbonic acid) for the CO bonds. On the basis of these R values, the normalization α constants equal to 88.09, 91.60, and 75.0 were used for CC, CN, and CO bonds, respectively [88].

**Polarizable Continuum Model (PCM)**

Model: PCM

Atomic radii: UA0 (Simple United Atom Topological Model)

Polarization charges: Total charges

Charge compensation: None

Solution method: Matrix inversion

Cavity: GePol (RMin=0.200 OFac=0.890)

Default sphere list used, NSphG= 9

Tesserae with average area of 0.200 Ang**2

1st derivatives: Analytical V*U(x)*V algorithm (CHGder, D1EAlg=0)

Cavity 1st derivative terms included

Solvent: Water

Eps = 78.390000

Eps(inf)= 1.776000

RSolv = 1.385000 Ang

**Table S1** Structures, electronic energies, and atom coordinates for neutral and ionized forms of 1-methylcytosine found at the DFT(B3LYP)/6-311+G(d,p) level

| Structure of neutral isomer | Energy (in Hartree) and atom coordinates (in Å) |
| --- | --- |
| **1**  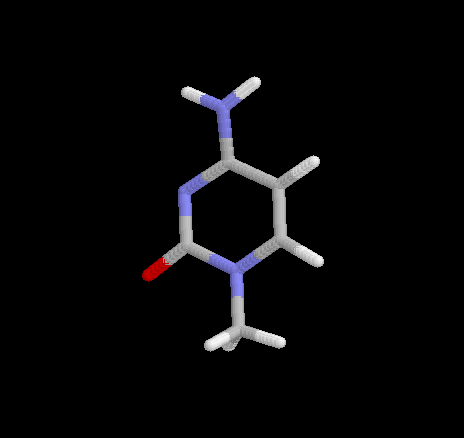 | scf done: -434.371692  C -1.204220 -0.964477 -0.043786  N -1.240561 0.344491 -0.175542  C 0.006169 -1.701068 0.170886  C 1.150525 -0.970284 0.231184  N 1.137131 0.375923 0.093661  C -0.094241 1.086841 -0.121416  O -0.040599 2.298314 -0.237131  C 2.360907 1.173186 0.156407  N -2.388137 -1.632766 -0.146696  H 0.022696 -2.776868 0.271802  H 2.119312 -1.429373 0.389448  H 2.496395 1.724058 -0.775067  H 2.293795 1.898422 0.968234  H 3.209161 0.508580 0.321476  H -2.462473 -2.594213 0.137875  H -3.227960 -1.075832 -0.176323 |

|  |  |
| --- | --- |
| **2a**  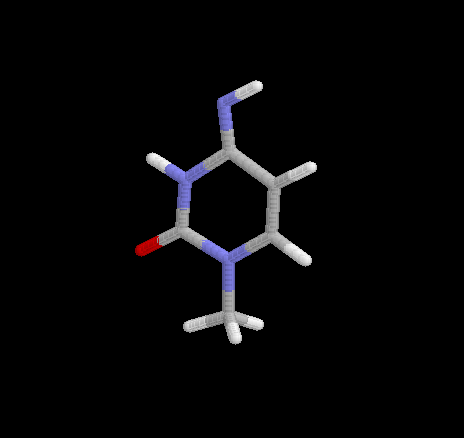 | scf done: -434.368144  C -1.294240 -1.092686 -0.051174  N -1.203957 0.308347 -0.042756  C 0.005127 -1.745412 -0.005016  C 1.128038 -1.003596 0.039610  N 1.127720 0.375762 0.045068  C -0.073606 1.095006 0.001141  O -0.118184 2.310609 0.001904  C 2.392007 1.111302 0.089922  N -2.464529 -1.610828 -0.096760  H 0.057540 -2.824636 -0.006697  H 2.110842 -1.457926 0.074724  H 2.995221 0.887253 -0.793366  H 2.160990 2.173079 0.106535  H 2.951810 0.848783 0.990765  H -2.409970 -2.626631 -0.098489  H -2.079553 0.814558 -0.074471 |
|  |  |
| **2b**  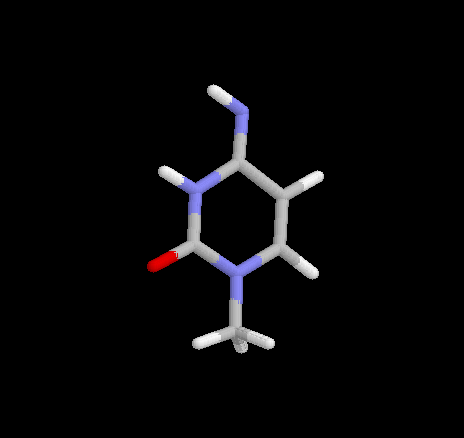 | scf done: -434.365377  C -1.296719 -1.098929 -0.055150  N -1.200005 0.308230 -0.045936  C 0.000515 -1.750967 -0.004974  C 1.125302 -1.017041 0.043084  N 1.132676 0.368281 0.048817  C -0.056576 1.090760 0.001735  O -0.107259 2.306757 0.001256  C 2.402845 1.093367 0.097473  N -2.391364 -1.761344 -0.101946  H 0.028027 -2.829830 -0.007560  H 2.106107 -1.474312 0.081128  H 3.006366 0.863007 -0.784004  H 2.182826 2.157470 0.113877  H 2.957486 0.824369 0.999614  H -2.051948 0.851935 -0.079790  H -3.212150 -1.156002 -0.1322868 |
|  |  |
| **3a**  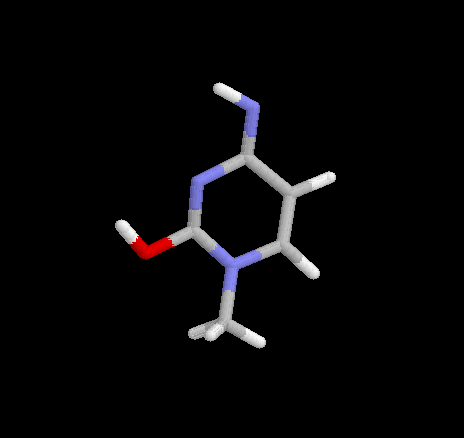 | scf done: -434.339765  C -1.305913 -1.086614 -0.036613  N -1.247720 0.319214 -0.138853  C -0.022285 -1.769039 0.131062  C 1.117153 -1.062450 0.186053  N 1.112122 0.328352 0.088697  C -0.112536 0.915919 -0.076942  O -0.042212 2.260070 -0.177612  C 2.348107 1.111990 0.120205  N -2.400154 -1.753713 -0.084701  H -0.014652 -2.846761 0.210723  H 2.093065 -1.511994 0.309789  H 2.530891 1.594802 -0.842034  H 2.297797 1.878611 0.893944  H 3.172874 0.436353 0.342327  H -3.184954 -1.112113 -0.204024  H -0.954213 2.564729 -0.288425 |

|  |  |
| --- | --- |
| **3b**  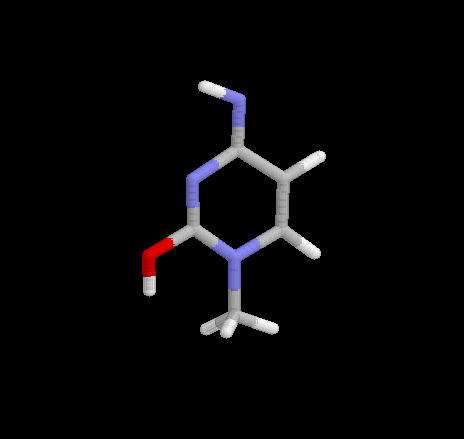 | scf done: -434.323692  C -1.585402 -0.629634 -0.009294  N -0.330329 -1.268321 -0.010034  C -1.569438 0.832822 -0.046301  C -0.401713 1.489749 -0.046551  N 0.816784 0.805321 -0.022237  C 0.744491 -0.578646 -0.008708  O 1.920942 -1.249664 0.023820  C 2.076711 1.533646 0.087504  N -2.697375 -1.267546 0.014318  H -2.509733 1.365216 -0.068924  H -0.323815 2.568437 -0.068673  H 2.732123 1.343518 -0.770095  H 1.860174 2.600513 0.096092  H 2.603727 1.289704 1.015026  H -2.509565 -2.270740 0.037012  H 2.668103 -0.673129 -0.165235 |
|  |  |
| **3c**  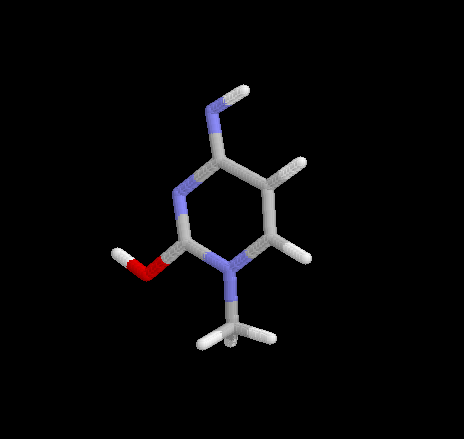 | scf done: -434.334238  C -1.539389 -0.719722 -0.058738  N -0.248888 -1.276851 -0.052850  C -1.589933 0.748145 -0.010077  C -0.456961 1.468796 0.045026  N 0.788243 0.855550 0.058180  C 0.779189 -0.516912 0.000091  O 2.020652 -1.046828 0.004130  C 2.034611 1.622630 0.095956  N -2.547990 -1.513153 -0.107112  H -2.547424 1.253165 -0.016546  H -0.441619 2.550103 0.084179  H 2.609078 1.479527 -0.821369  H 1.781663 2.677162 0.198140  H 2.647572 1.318829 0.945287  H 1.894586 -2.006186 -0.036814  H -3.413731 -0.974420 -0.106986 |
|  |  |
| **3d**  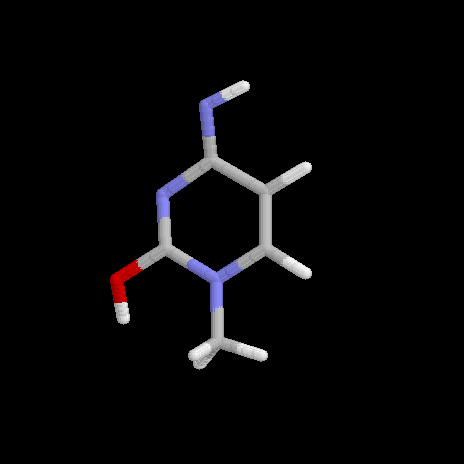 | scf done: -434.316625  C -1.579982 -0.647473 -0.000997  N -0.325404 -1.277875 -0.004948  C -1.555401 0.820038 -0.068057  C -0.385989 1.477601 -0.069899  N 0.826825 0.796900 -0.029300  C 0.747108 -0.592425 -0.009213  O 1.925868 -1.261158 0.026948  C 2.087035 1.522956 0.093389  N -2.632309 -1.381274 0.052092  H -2.484135 1.375019 -0.106366  H -0.311324 2.556675 -0.106231  H 2.746273 1.341672 -0.763208  H 1.871876 2.590150 0.114092  H 2.609131 1.265363 1.019482  H 2.666536 -0.694914 -0.211947  H -3.465703 -0.793137 0.042344 |

| Structure of radical cation |  |
| --- | --- |
| **1**^+•^  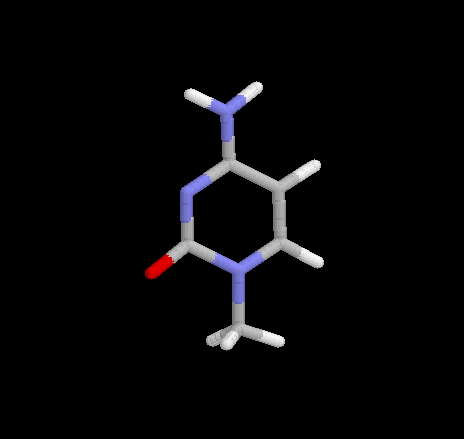 | scf done: -434.064894  C -1.246301 -0.957818 -0.030921  N -1.261257 0.354047 -0.171989  C -0.007451 -1.678124 0.175881  C 1.184094 -0.951349 0.226236  N 1.171547 0.361755 0.086704  C -0.127461 1.078848 -0.128241  O -0.034627 2.277077 -0.245913  C 2.397126 1.173149 0.132109  N -2.400082 -1.629781 -0.081676  H 0.019074 -2.754720 0.291190  H 2.138556 -1.442665 0.379567  H 2.497953 1.716035 -0.808234  H 2.310761 1.900864 0.939610  H 3.250625 0.517954 0.292675  H -2.453605 -2.631579 0.018266  H -3.257838 -1.112886 -0.227421 |
|  |  |
| **2a**^+•^  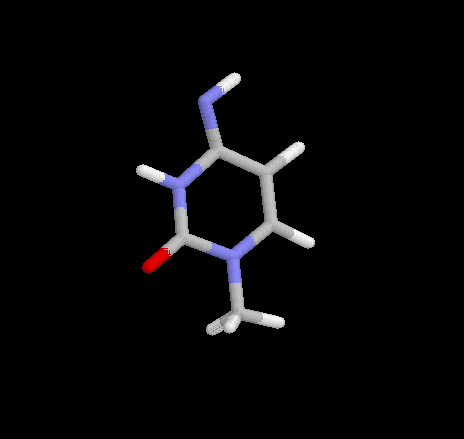 | scf done: -434.065227  C -1.273748 -1.064891 -0.030994  N -1.215308 0.311586 -0.156221  C -0.039292 -1.737143 0.161564  C 1.137621 -0.994507 0.213030  N 1.137846 0.329410 0.086768  C -0.085829 1.096731 -0.113428  O -0.050113 2.284615 -0.222651  C 2.374697 1.129715 0.139079  N -2.469856 -1.604731 -0.103794  H -0.009861 -2.812627 0.267341  H 2.099205 -1.473391 0.358667  H 2.477795 1.681754 -0.795895  H 2.296009 1.848195 0.955866  H 3.219977 0.463011 0.290148  H -2.436873 -2.621299 -0.003162  H -2.094814 0.804142 -0.294532 |
|  |  |
| **2b**^+•^  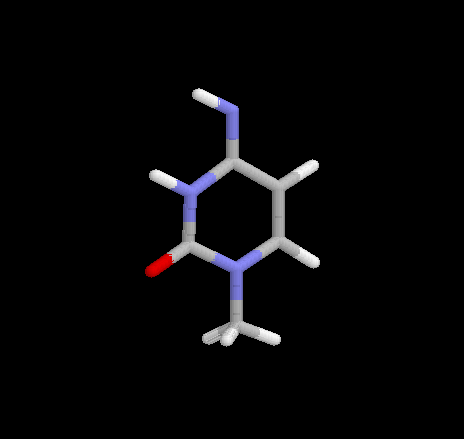 | scf done: -434.063617  C -1.291351 -1.059421 -0.019793  N -1.217182 0.324409 -0.151195  C -0.052794 -1.734494 0.168208  C 1.132402 -1.006668 0.210870  N 1.145070 0.318153 0.079696  C -0.070826 1.092694 -0.115951  O -0.026624 2.280315 -0.230142  C 2.390677 1.106269 0.122174  N -2.386629 -1.769207 -0.055743  H -0.059231 -2.809915 0.275065  H 2.089445 -1.495121 0.352816  H 2.495409 1.653337 -0.815423  H 2.325380 1.827562 0.937514  H 3.228767 0.430069 0.270692  H -2.068066 0.861950 -0.287761  H -3.236175 -1.214160 -0.194129 |

|  |  |
| --- | --- |
| **3a**^+•^  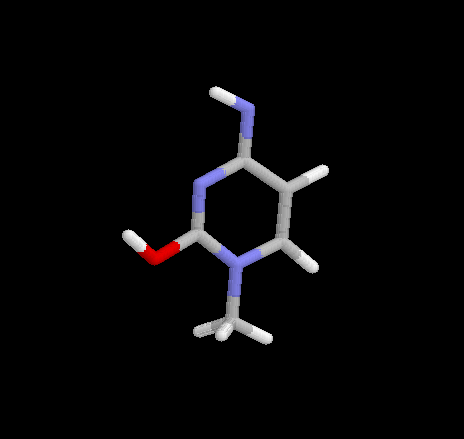 | scf done: -434.055322  C -1.261466 -1.058370 -0.030114  N -1.255129 0.300617 -0.146921  C -0.041044 -1.767739 0.148715  C 1.123972 -1.043843 0.198512  N 1.101148 0.308331 0.079470  C -0.116428 0.930369 -0.091713  O -0.032842 2.240916 -0.196871  C 2.344188 1.114348 0.129744  N -2.401635 -1.759488 -0.079392  H -0.044501 -2.844815 0.241378  H 2.097275 -1.497051 0.331543  H 2.452412 1.665587 -0.803928  H 2.282247 1.818075 0.959338  H 3.184069 0.438716 0.269239  H -3.194575 -1.120682 -0.210714  H -0.920213 2.618033 -0.314849 |
|  |  |
| **3b**^+•^  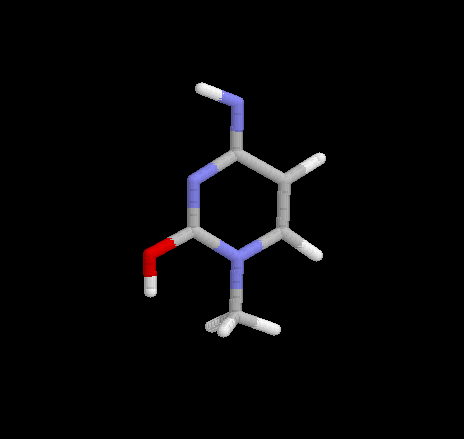 | scf done: -434.039250  C -1.531667 -0.604503 -0.023191  N -0.345102 -1.259197 -0.009895  C -1.578443 0.818759 -0.017756  C -0.388957 1.491356 0.001112  N 0.794532 0.809409 0.014148  C 0.765048 -0.572093 0.007910  O 1.890968 -1.257225 0.020008  C 2.079221 1.542863 0.034352  N -2.695043 -1.279222 -0.041659  H -2.526358 1.338419 -0.028303  H -0.317752 2.570604 0.006665  H 2.659461 1.302700 -0.859399  H 1.872781 2.610026 0.036789  H 2.635999 1.294225 0.940628  H -2.500920 -2.288039 -0.043177  H 2.697123 -0.725359 0.034015 |
|  |  |
| **3c**^+•^  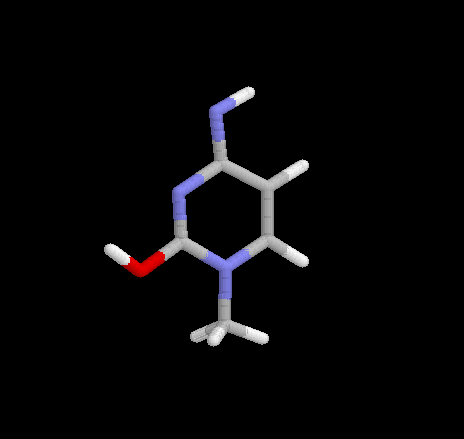 | scf done: -434.049200  C -1.497986 -0.683025 -0.054288  N -0.271831 -1.271445 -0.057638  C -1.599094 0.738522 0.002991  C -0.439564 1.470967 0.053631  N 0.767655 0.852841 0.048816  C 0.791704 -0.523113 -0.008123  O 2.004875 -1.035524 -0.008532  C 2.036321 1.617214 0.102655  N -2.544657 -1.526248 -0.107417  H -2.558065 1.239254 0.007109  H -0.425327 2.551930 0.098875  H 2.626156 1.395112 -0.786194  H 1.796389 2.676539 0.142811  H 2.595527 1.320865 0.989639  H 1.947082 -2.004992 -0.049960  H -3.427217 -1.003930 -0.101544 |

|  |  |
| --- | --- |
| **3d**^+•^  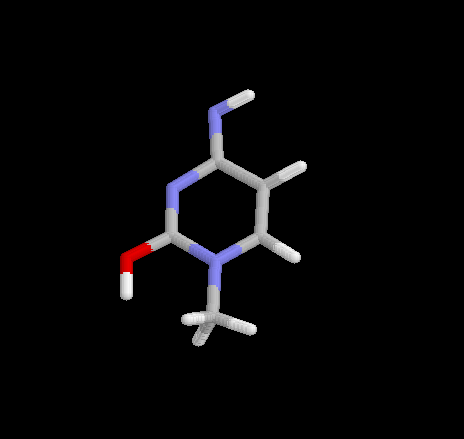 | scf done: -434.031589  C -1.529298 -0.614346 -0.021038  N -0.343569 -1.264426 -0.009962  C -1.565322 0.813384 -0.013717  C -0.374527 1.483102 0.002545  N 0.806498 0.800007 0.013267  C 0.768001 -0.581397 0.007373  O 1.890084 -1.272125 0.018717  C 2.093477 1.529196 0.030860  N -2.621644 -1.407484 -0.041848  H -2.498131 1.361377 -0.021346  H -0.302392 2.562470 0.008372  H 2.671547 1.285138 -0.863237  H 1.891126 2.597179 0.032312  H 2.650635 1.278785 0.936418  H 2.699390 -0.745121 0.032124  H -3.475823 -0.839140 -0.040716 |
|  |  |
| **4a**^+•^  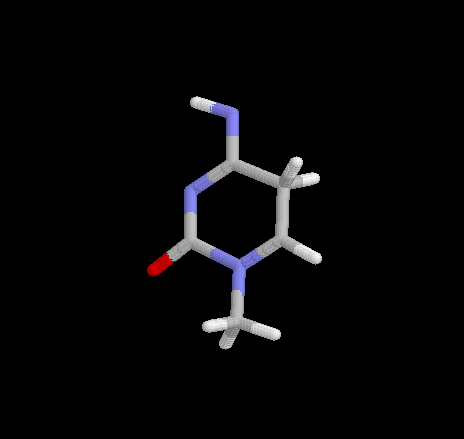 | scf done: -434.010782  C -1.313457 -0.959084 -0.071905  N -1.326709 0.349916 0.141373  C -0.044190 -1.746532 -0.282684  C 1.181340 -0.928966 -0.223481  N 1.154216 0.340083 -0.015476  C -0.162784 1.082528 0.182744  O -0.082618 2.255797 0.361448  C 2.371295 1.179903 0.047372  N -2.449718 -1.650755 -0.110298  H 2.150949 -1.403337 -0.359162  H 3.243083 0.547325 -0.106079  H 2.306904 1.945805 -0.724500  H 2.410559 1.664763 1.022024  H -3.239688 -1.012503 0.045699  H -0.077600 -2.273027 -1.247392  H 0.028990 -2.557201 0.456351 |
|  |  |
| **4b**^+•^  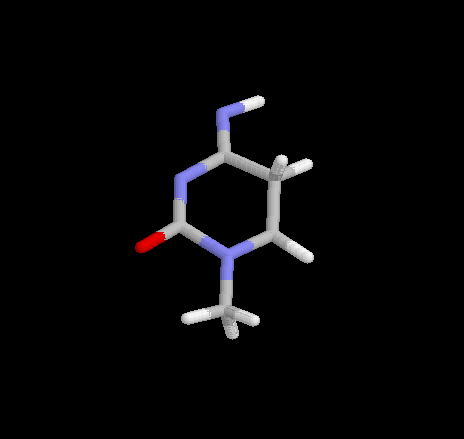 | scf done: -434.001477  C -1.302357 -0.976367 0.085227  N -1.305279 0.340103 0.280927  C -0.053476 -1.699702 -0.394846  C 1.184055 -0.900793 -0.230406  N 1.148042 0.362385 -0.003347  C -0.194180 1.097553 0.023882  O -0.154979 2.273402 -0.146554  C 2.351314 1.208919 0.148335  N -2.448299 -1.591200 0.342086  H 2.155955 -1.383733 -0.308276  H 3.235787 0.576383 0.110095  H 2.361797 1.945163 -0.653999  H 2.290671 1.727858 1.104946  H -0.148916 -1.956300 -1.462122  H 0.071982 -2.659034 0.119248  H -2.400826 -2.594233 0.131726 |

|  |  |
| --- | --- |
| **5a**^+•^  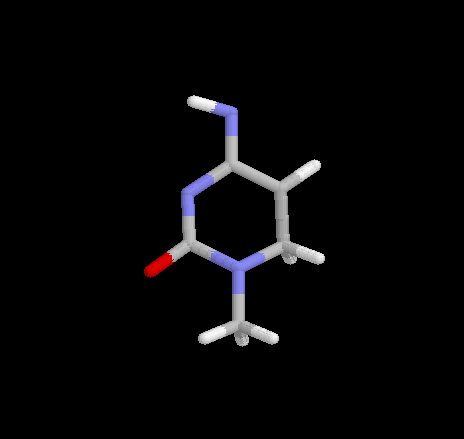 | scf done: -433.934134  C -1.296572 -1.022748 -0.035565  N -1.242460 0.229244 0.470292  C -0.081097 -1.656837 -0.450284  C 1.162644 -0.948603 -0.310722  N 1.106520 0.415060 0.077224  C -0.129752 1.058167 0.205852  O -0.263486 2.249923 0.131547  C 2.327758 1.232715 0.072708  N -2.417492 -1.744470 -0.115629  H 3.174363 0.615804 0.373423  H 2.503143 1.656045 -0.920290  H 2.195420 2.052989 0.777116  H 1.680480 -1.563434 0.475216  H 1.824563 -1.126687 -1.178978  H -0.085049 -2.697548 -0.761520  H -3.208903 -1.211556 0.267518 |
|  |  |
| **5b**^+•^  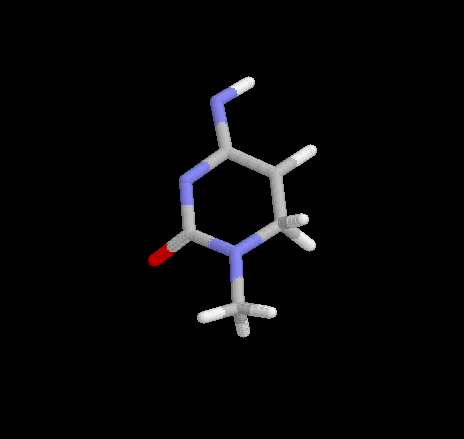 | scf done: -433.923382  C -1.162997 -1.164134 -0.203701  C -0.469142 -0.832953 -1.409363  C 0.440340 0.293759 -1.430607  N 0.732733 0.918183 -0.189589  C 0.292422 0.307367 1.000498  N -0.888054 -0.439691 0.908089  C 1.729853 1.990851 -0.110435  O 0.867348 0.444949 2.046091  H 1.362854 0.019392 -1.982183  H 1.522393 2.589151 0.776423  H 2.735870 1.571225 -0.011610  H 1.667197 2.607485 -1.006747  N -2.164557 -2.053067 -0.113417  H -2.263151 -2.595988 -0.977985  H -0.689506 -1.330161 -2.349224  H -0.018147 1.031988 -2.131338 |
|  |  |
| Structure of radical anion |  |
| **1**^-•^  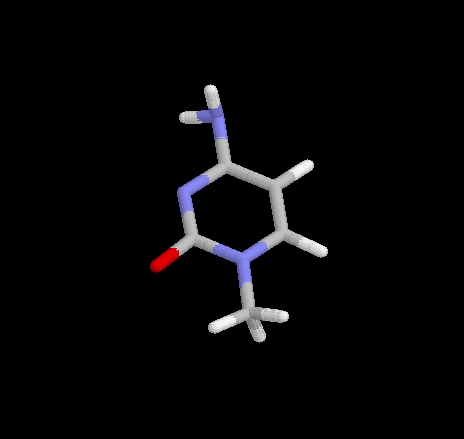 | scf done: -434.357899  C -1.947100 -0.848210 -0.058500  N -2.046300 0.533800 -0.145730  C -0.705740 -1.519320 0.017260  C 0.470220 -0.758800 -0.036210  N 0.419510 0.619440 -0.204970  C -0.889760 1.263550 -0.176460  O -0.871700 2.531130 -0.209050  C 1.597630 1.381930 0.052530  N -3.150170 -1.639840 -0.027490  H -0.640550 -2.607190 0.115090  H 1.463510 -1.218480 0.042040  H 2.420590 1.058270 -0.641820  H 1.382550 2.470950 -0.113970  H 1.969020 1.245400 1.106430  H -3.546040 -1.643150 -0.953210  H -3.824130 -1.195520 0.573030 |

|  |  |
| --- | --- |
| **2a**^-•^  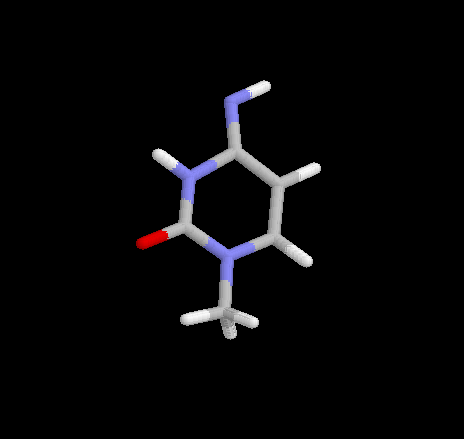 | scf done: -434.362894  C -1.265671 -1.124264 -0.037211  N -1.188964 0.301458 0.042287  C -0.015189 -1.760592 0.006256  C 1.186895 -1.028406 0.163668  N 1.129975 0.393380 -0.004478  C -0.058161 1.081360 -0.020900  O -0.131245 2.319265 -0.071026  C 2.365524 1.134933 0.088567  N -2.502805 -1.585284 -0.126526  H 0.023460 -2.843198 -0.020853  H 2.147730 -1.453109 -0.110356  H 3.062754 0.813043 -0.696560  H 2.150174 2.195056 -0.027618  H 2.847314 0.958974 1.062500  H -2.466288 -2.603226 -0.159142  H -2.063015 0.793270 -0.061027 |
|  |  |
| **2b**^-•^  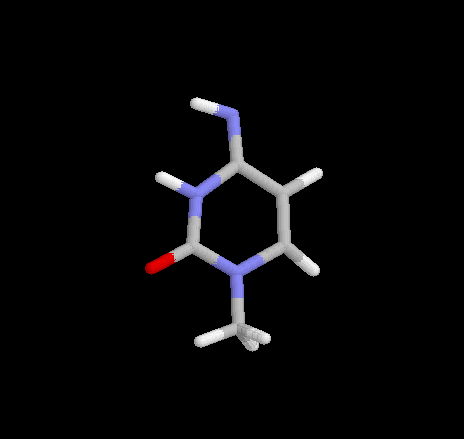 | scf done: -434.357634  C -1.263846 -1.141546 -0.053046  N -1.175061 0.295473 -0.184118  C -0.012420 -1.767741 0.002948  C 1.193523 -1.038379 -0.082561  N 1.131748 0.385676 0.072751  C -0.046531 1.074189 0.003696  O -0.132348 2.311004 0.073739  C 2.374364 1.120457 0.112828  N -2.447353 -1.732925 -0.029534  H 0.012826 -2.848035 0.055398  H 2.138315 -1.462891 0.238515  H 2.965240 0.915944 -0.792421  H 2.154245 2.184082 0.176151  H 2.971238 0.817021 0.983373  H -2.030336 0.820409 -0.081938  H -3.188627 -1.034007 -0.085869 |
|  |  |
| **3a**^-•^  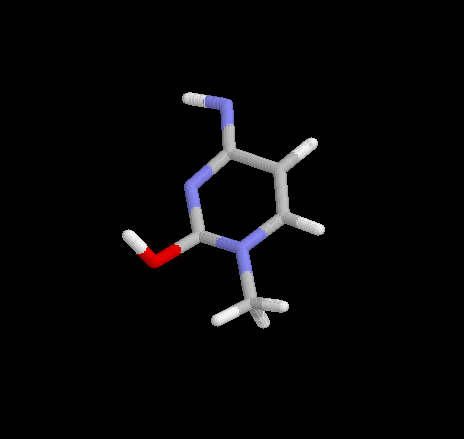 | scf done: -434.317575  C -1.503517 -0.717709 -0.130874  N -0.260894 -1.280762 -0.135862  C -1.585664 0.755099 -0.073742  C -0.459986 1.472747 0.122085  N 0.780624 0.877581 0.227840  C 0.840070 -0.493013 -0.216147  O 2.023858 -1.115937 0.227976  C 1.957154 1.697579 0.000986  N -2.630092 -1.389719 -0.152099  H -2.557360 1.229139 -0.113167  H -0.467890 2.555036 0.213457  H 2.143401 1.860614 -1.075602  H 1.823609 2.666183 0.492003  H 2.829475 1.201867 0.424810  H -2.386631 -2.382435 -0.191130  H 1.708722 -1.940832 0.632808 |

|  |  |
| --- | --- |
| **3b**^-•^  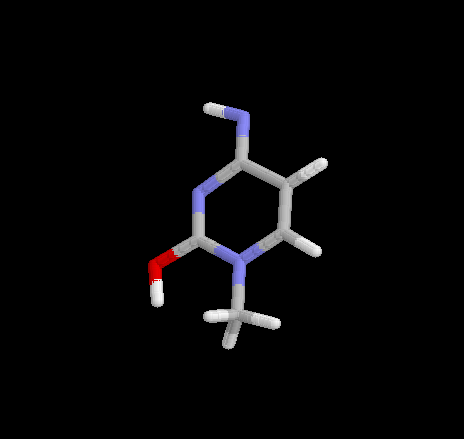 | scf done: -434.312616  C -0.969708 0.443730 -1.328220  C -1.763261 -0.042545 -0.207884  C -1.147507 -0.431885 0.921758  N 0.232556 -0.378945 1.056348  C 0.943402 0.089216 -0.030746  N 0.415826 0.479928 -1.144386  C 0.872602 -0.851819 2.293120  O 2.279170 0.141905 0.060634  H -2.840261 -0.086199 -0.297636  H -1.673361 -0.802003 1.790637  H 1.530572 -1.707903 2.105349  H 1.468176 -0.063348 2.766468  H 0.093825 -1.162501 3.000586  H 2.630902 -0.110942 0.941804  N -1.540620 0.818500 -2.427133  H -0.810722 1.121091 -3.074251 |
|  |  |
| **3c**^-•^  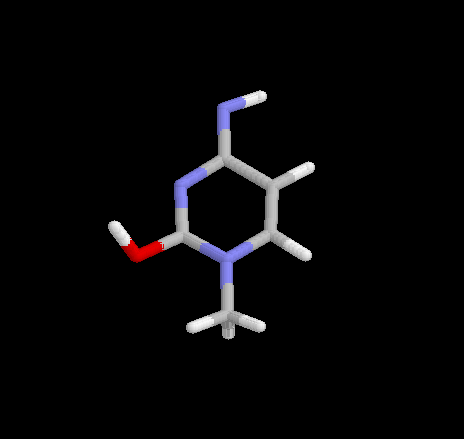 | scf done: -434.310151  C -1.491015 -0.748483 -0.131236  N -0.247755 -1.293106 -0.143950  C -1.572605 0.734757 -0.084582  C -0.451553 1.458961 0.125970  N 0.786364 0.868671 0.230239  C 0.843052 -0.497695 -0.226912  O 2.035276 -1.117263 0.201169  C 1.966247 1.690588 0.029328  N -2.568734 -1.503371 -0.135192  H -2.537977 1.226772 -0.129829  H -0.466081 2.541125 0.220813  H 2.178374 1.845220 -1.043559  H 1.816534 2.661994 0.509809  H 2.829085 1.200524 0.478390  H 1.728178 -1.981020 0.519441  H -3.384204 -0.890628 -0.097497 |
|  |  |
| **3d**^-•^  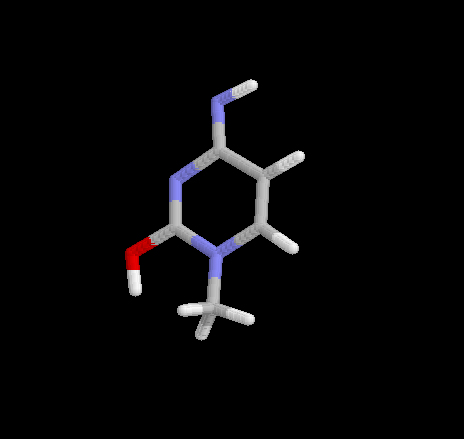 | scf done: -434.305505  C -0.724844 0.331440 -1.505502  C 0.727212 0.481207 -1.522976  C 1.445477 0.244949 -0.407066  N 0.839244 -0.174416 0.765949  C -0.539865 -0.299702 0.732758  N -1.283454 -0.090411 -0.296542  C 1.621457 -0.254070 2.008403  O -1.155615 -0.655689 1.874030  H 1.233677 0.790365 -2.430313  H 2.522002 0.331902 -0.360834  H 1.296786 0.499320 2.737289  H 1.541569 -1.241910 2.474266  H 2.677301 -0.074302 1.775720  H -0.535536 -0.963075 2.565908  N -1.515158 0.554174 -2.509637  H -0.951929 0.854843 -3.306366 |

|  |  |
| --- | --- |
| **4a**^-•^  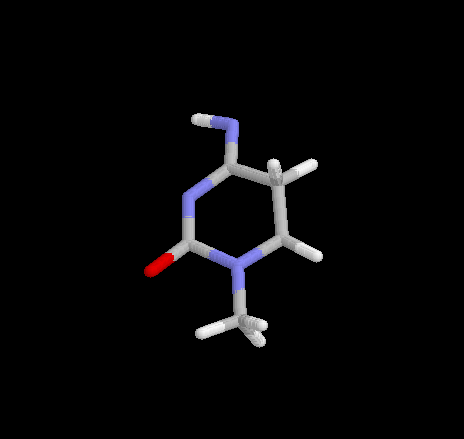 | scf done: -434.363731  C -1.312953 -0.980480 0.043473  N -1.296037 0.378309 -0.042861  C -0.036616 -1.753625 -0.270875  C 1.157862 -0.987665 0.177198  N 1.110017 0.379607 -0.001181  C -0.156691 1.080551 -0.127568  O -0.091065 2.311519 -0.276985  C 2.320017 1.150322 0.194647  N -2.369773 -1.684231 0.318665  H 2.147751 -1.434077 0.176770  H 3.115720 0.793605 -0.472595  H 2.093665 2.191163 -0.026308  H 2.680256 1.069058 1.231436  H -3.139093 -1.024186 0.461550  H -0.021931 -1.916028 -1.368090  H -0.087015 -2.742108 0.189503 |
|  |  |
| **4b**^-•^  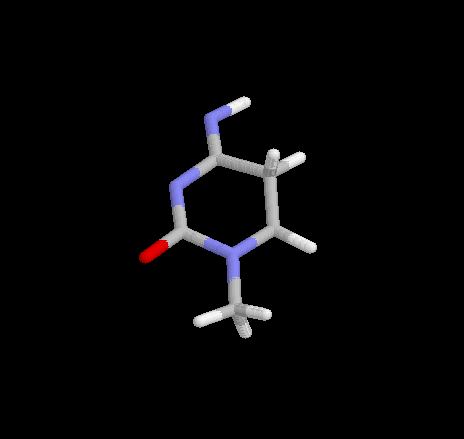 | scf done: -434.354144  C -1.325833 -0.964182 0.080040  N -1.307221 0.388355 -0.004744  C -0.045563 -1.720414 -0.313539  C 1.157194 -0.970166 0.137312  N 1.103429 0.396841 -0.015879  C -0.174817 1.090482 -0.126989  O -0.110881 2.318544 -0.296583  C 2.309336 1.172757 0.181162  N -2.410946 -1.605032 0.411673  H 2.145806 -1.419462 0.117742  H 3.101706 0.835260 -0.500204  H 2.071233 2.214915 -0.020932  H 2.680517 1.076857 1.212865  H -0.065624 -1.827831 -1.417859  H -0.050619 -2.733704 0.099425  H -2.214705 -2.606404 0.396362 |
|  |  |
| **5a**^-•^  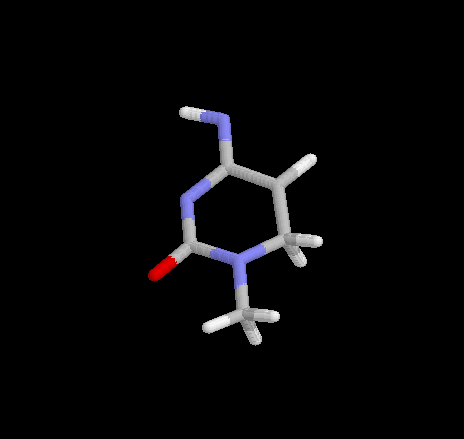 | scf done: -434.357073  C -1.298030 -1.019057 -0.065366  N -1.314601 0.321447 0.211311  C -0.057614 -1.723047 -0.222245  C 1.222807 -0.997543 -0.050026  N 1.050263 0.430494 -0.233866  C -0.188147 1.056028 0.162124  O -0.145978 2.276582 0.391369  C 2.266679 1.192909 -0.067817  N -2.400077 -1.747816 -0.175721  H 3.019971 0.854887 -0.792390  H 2.046773 2.246211 -0.225708  H 2.700326 1.082127 0.944212  H 1.651517 -1.213370 0.960667  H 1.981016 -1.365552 -0.760857  H -0.069521 -2.796051 -0.371058  H -3.185521 -1.105531 -0.037913 |

|  |  |
| --- | --- |
| **5b**^-•^  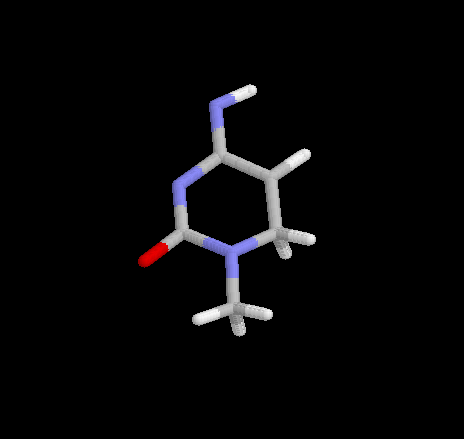 | scf done: -434.346825  C -1.319548 -0.994745 -0.044275  N -1.319385 0.337106 0.240514  C -0.072724 -1.699735 -0.211950  C 1.212854 -0.979054 -0.050698  N 1.039493 0.442712 -0.262480  C -0.191970 1.065636 0.173714  O -0.136105 2.282371 0.416985  C 2.257948 1.206780 -0.116298  N -2.472072 -1.654668 -0.139930  H 3.006273 0.853933 -0.838965  H 2.038794 2.257337 -0.292255  H 2.696867 1.115862 0.895467  H -2.253737 -2.635249 -0.328397  H 1.637839 -1.179181 0.965259  H 1.969112 -1.364178 -0.754334  H -0.061931 -2.776832 -0.352349 |
|  |  |
| Structure of protonated form |  |
| **MCH**^+^  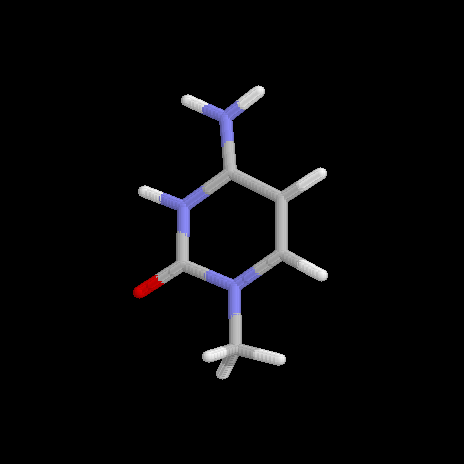 | scf done: -434.753040  C -1.451228 -0.961642 -0.208927  N -1.374856 0.330648 -0.614476  N 0.725551 0.658434 0.401788  C 0.674597 -0.626785 0.818465  C -0.370250 -1.465401 0.546447  C 1.866227 1.548186 0.707711  N -2.519591 -1.687678 -0.532390  H 1.525271 -0.969407 1.394294  H -0.366875 -2.485029 0.900694  H 2.316215 1.893916 -0.222021  H 1.513374 2.408851 1.274452  H -3.288176 -1.317938 -1.073114  H -2.589006 -2.650254 -0.236898  H 2.593288 0.987596 1.290652  H -2.118013 0.755036 -1.160525  C -0.313562 1.222352 -0.351689  O -0.322575 2.353394 -0.747132 |
|  |  |
| Structure of deprotonated form |  |
| **MC-H**^+^  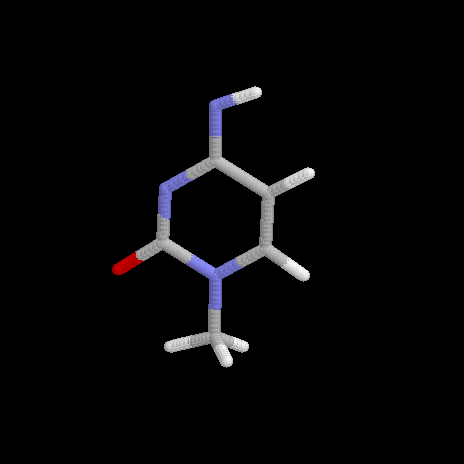 | scf done: -433.792442  C 1.018190 -0.372810 0.041512  N -0.188026 -1.002691 0.211966  C -1.343357 -0.348569 0.317885  N -1.325499 1.114227 0.263331  C -0.139624 1.768249 0.079737  C 1.025084 1.098576 -0.032686  C -2.565351 1.857917 0.341143  N 2.118710 -1.080558 -0.050330  O -2.463960 -0.864335 0.465366  H -0.195067 2.853375 0.034394  H 1.955493 1.638069 -0.171672  H -3.351486 1.143793 0.580078  H -2.517136 2.627443 1.123017  H 2.901941 -0.436779 -0.173410  H -2.809030 2.347363 -0.613033 |
|  |  |

| Structure of neutral radical |  |
| --- | --- |
| **MCH**^•^  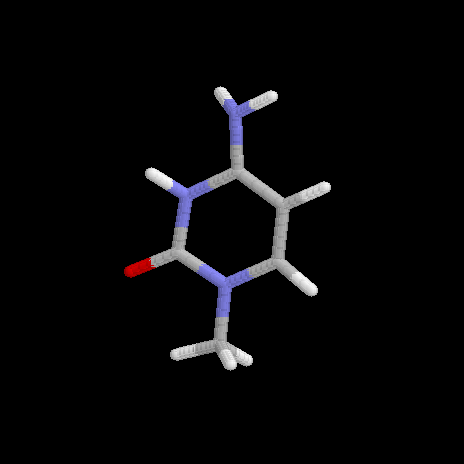 | scf done: -434.937904  C 1.047716 -0.285710 0.029781  N -0.178169 -0.934324 0.246464  C -1.413978 -0.292147 0.308624  N -1.356247 1.087840 0.275564  C -0.148036 1.787249 0.084326  C 1.049518 1.087789 -0.028952  C -2.598305 1.845629 0.359157  N 2.133808 -1.154148 -0.137710  O -2.450007 -0.931518 0.389209  H -0.223691 2.862166 0.049924  H 1.978082 1.629303 -0.150701  H -3.412738 1.155691 0.562945  H -2.530527 2.582474 1.164787  H 2.988575 -0.672554 -0.381708  H 2.296651 -1.770147 0.655249  H -0.245834 -1.921813 0.044689  H -2.793368 2.366028 -0.584080 |
|  |  |
| **MC-H**^•^  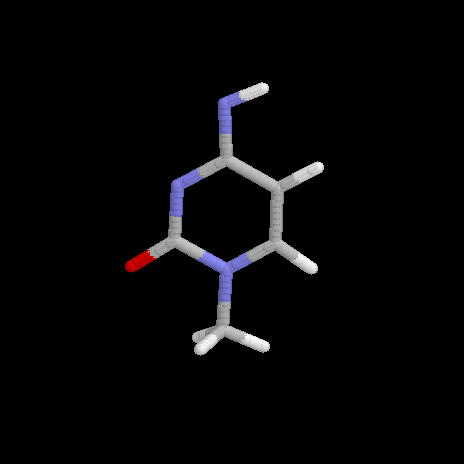 | scf done: -433.686631  C -1.684477 -0.714740 0.006832  N -1.714449 0.618821 0.011655  C -0.545590 1.346620 0.008501  N 0.685649 0.610299 0.002664  C 0.705426 -0.745297 -0.002539  C -0.451243 -1.459234 -0.001025  C 1.916478 1.399715 0.000771  N -2.903247 -1.321960 0.010249  O -0.494951 2.561375 0.009980  H 1.682548 -1.213138 -0.007753  H -0.438766 -2.539941 -0.005286  H 1.943700 2.042213 0.881915  H 2.772718 0.726119 -0.000013  H 1.941791 2.041777 -0.880816  H -2.761376 -2.337768 0.005494 |
|  |  |
| Structure of transition state |  |
| **TS**  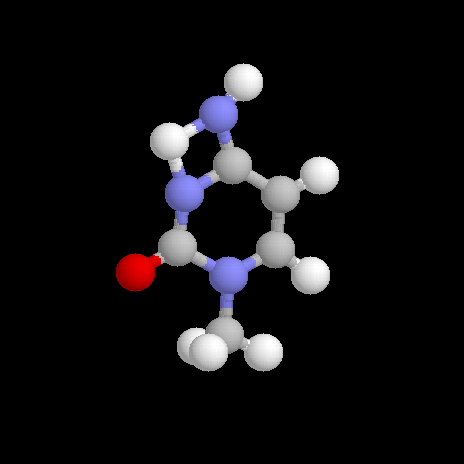 | scf done: -434.302033  C -1.127643 -1.156643 -0.000400  N -1.211942 0.199603 -0.155855  C 0.135668 -1.780909 0.206804  C 1.207218 -0.937904 0.234778  N 1.106125 0.407609 0.078686  C -0.163987 1.058500 -0.132180  O -0.201472 2.265869 -0.266363  C 2.282424 1.278235 0.115245  N -2.382591 -1.541097 -0.097955  H 0.251953 -2.846149 0.334457  H 2.211881 -1.314943 0.386737  H 2.378303 1.820873 -0.825848  H 2.184934 2.008857 0.918978  H 3.168842 0.666388 0.279400  H -2.682248 -2.503530 -0.027489  H -2.485111 -0.188926 -0.254940 |

|  |  |
| --- | --- |
| **TS**^+•^  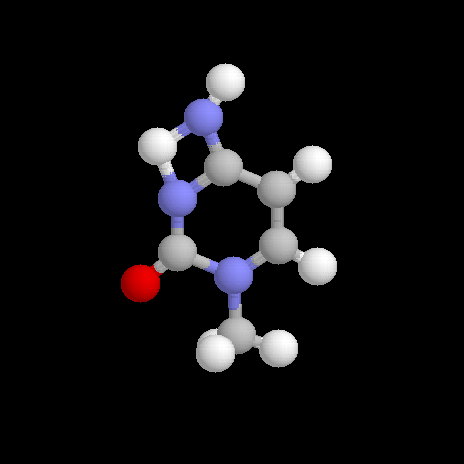 | scf done: -433.993251  C -1.113643 -1.127320 -0.005791  N -1.237043 0.214228 -0.157306  C 0.123609 -1.785073 0.196934  C 1.238310 -0.942742 0.231827  N 1.121782 0.372157 0.083903  C -0.200440 1.083642 -0.132758  O -0.186135 2.268707 -0.252027  C 2.287158 1.262344 0.119311  N -2.373353 -1.537990 -0.101330  H 0.217200 -2.854798 0.317590  H 2.236006 -1.340263 0.381545  H 2.336322 1.812754 -0.822419  H 2.147091 1.991338 0.920030  H 3.189577 0.676829 0.277284  H -2.691237 -2.503667 -0.036899  H -2.535556 -0.215725 -0.254917 |
|  |  |
| **TS**^-•^  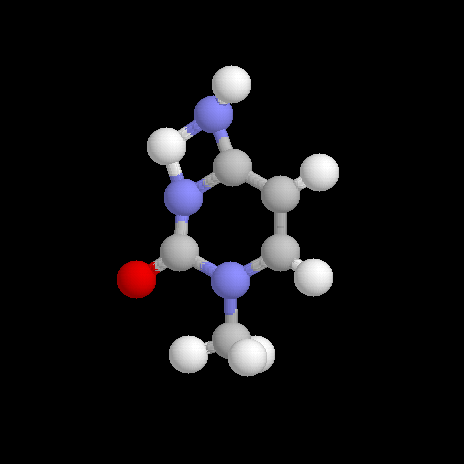 | scf done: -434.303914  C -1.094617 -1.193711 -0.021776  N -1.212867 0.203342 0.029089  C 0.140963 -1.801299 -0.041451  C 1.283063 -0.963426 0.020581  N 1.104881 0.443875 -0.030058  C -0.168444 1.060638 0.025522  O -0.265577 2.296208 0.046871  C 2.282911 1.269689 0.080275  N -2.423364 -1.549287 -0.019068  H 0.254069 -2.878253 -0.064502  H 2.279979 -1.306278 -0.230034  H 2.986877 1.050665 -0.736007  H 1.978997 2.313176 0.026646  H 2.806542 1.088912 1.033444  H -2.680330 -2.384357 -0.534736  H -2.445331 -0.170394 -0.108425 |

**Table S2** Mulliken atomic charges and atomic spin populations found in the gas phase for heavy atoms in neutral and ionized **MC** isomers^a^

| Charge |  |  |  | Spin density |  |
| --- | --- | --- | --- | --- | --- |
| Neutral | Radical cation | Radical anion | Isomer-atom | Radical cation | Radical anion |
| -0.09 | -0.03 | -0.01 | **1**-N1 | 0.32 | 0.03 |
| 0.21 | 0.20 | 0.21 | **1**-C2 | -0.10 | -0.01 |
| -0.28 | -0.18 | -0.37 | **1**-N3 | 0.12 | 0.14 |
| 0.02 | 0.03 | -0.08 | **1**-C4 | -0.11 | 0.20 |
| -0.09 | 0.11 | -0.10 | **1**-C5 | 0.60 | -0.08 |
| -0.05 | -0.04 | -0.40 | **1**-C6 | -0.11 | 0.69 |
| -0.37 | -0.16 | -0.48 | **1**-O7 | 0.27 | -0.01 |
| -0.35 | -0.34 | -0.37 | **1**-N8 | 0.01 | 0.02 |
| -0.23 | -0.23 | -0.30 | **1**-C9 | -0.02 | 0.00 |
| -0.19 | -0.10 | -0.05 | **2a**-N1 | 0.25 | -0.02 |
| 0.44 | 0.40 | 0.36 | **2a**-C2 | -0.02 | 0.02 |
| -0.38 | -0.34 | -0.32 | **2a**-N3 | 0.04 | 0.03 |
| -0.19 | -0.21 | -0.30 | **2a**-C4 | -0.19 | 0.12 |
| -0.05 | 0.10 | -0.15 | **2a**-C5 | 0.41 | 0.23 |
| 0.03 | 0.08 | -0.20 | **2a**-C6 | -0.04 | 0.47 |
| -0.37 | -0.22 | -0.48 | **2a**-O7 | -0.01 | 0.00 |
| -0.35 | -0.14 | -0.54 | **2a**-N8 | 0.59 | 0.17 |
| -0.27 | -0.23 | -0.28 | **2a**-C9 | -0.01 | -0.01 |
| -0.18 | -0.09 | -0.04 | **2b**-N1 | 0.27 | -0.02 |
| 0.41 | 0.38 | 0.29 | **2b**-C2 | -0.02 | 0.03 |
| -0.40 | -0.35 | -0.31 | **2b**-N3 | 0.02 | 0.02 |
| -0.07 | -0.04 | -0.37 | **2b**-C4 | -0.19 | 0.17 |
| -0.07 | 0.02 | -0.03 | **2b**-C5 | 0.44 | 0.21 |
| -0.01 | 0.04 | -0.23 | **2b**-C6 | -0.02 | 0.42 |
| -0.37 | -0.23 | -0.48 | **2b**-O7 | 0.00 | 0.01 |
| -0.34 | -0.15 | -0.48 | **2b**-N8 | 0.52 | 0.18 |
| -0.25 | -0.22 | -0.26 | **2b**-C9 | -0.01 | -0.01 |
| -0.11 | -0.06 | 0.23 | **3a**-N1 | 0.17 | -0.15 |
| 0.27 | 0.28 | -0.48 | **3a**-C2 | -0.06 | 1.05 |
| -0.27 | -0.14 | -0.30 | **3a**-N3 | 0.16 | 0.08 |
| -0.18 | -0.16 | -0.04 | **3a**-C4 | -0.17 | -0.15 |
| -0.05 | 0.00 | -0.30 | **3a**-C5 | 0.24 | 0.30 |
| -0.05 | 0.02 | 0.04 | **3a**-C6 | -0.01 | -0.20 |
| -0.27 | -0.22 | -0.22 | **3a**-O7 | -0.01 | -0.10 |
| -0.37 | -0.13 | -0.46 | **3a**-N8 | 0.71 | 0.05 |
| -0.22 | -0.22 | -0.39 | **3a**-C9 | -0.01 | 0.08 |
| -0.05 | 0.01 | 0.16 | **3b**-N1 | 0.16 | 0.48 |
| 0.19 | 0.21 | -0.27 | **3b**-C2 | -0.06 | -0.03 |
| -0.24 | -0.13 | -0.26 | **3b**-N3 | 0.18 | 1.24 |
| -0.10 | -0.09 | -0.83 | **3b**-C4 | -0.16 | 0.72 |
| -0.07 | -0.02 | -1.32 | **3b**-C5 | 0.20 | -0.54 |
| -0.05 | 0.01 | 0.51 | **3b**-C6 | 0.00 | -0.21 |
| -0.22 | -0.15 | 0.22 | **3b**-O7 | -0.01 | -0.24 |
| -0.36 | -0.11 | -0.41 | **3b**-N8 | 0.73 | -0.02 |
| -0.32 | -0.32 | -0.04 | **3b**-C9 | -0.01 | -0.45 |
| -0.12 | -0.06 | 0.22 | **3c**-N1 | 0.17 | -0.14 |
| 0.30 | 0.31 | -0.37 | **3c**-C2 | -0.06 | 0.95 |
| -0.20 | -0.09 | -0.23 | **3c**-N3 | 0.16 | 0.08 |
| -0.30 | -0.31 | -0.10 | **3c**-C4 | -0.17 | -0.12 |
| -0.07 | 0.05 | -0.35 | **3c**-C5 | 0.23 | 0.14 |
| 0.00 | 0.04 | 0.03 | **3c**-C6 | -0.02 | -0.06 |
| -0.27 | -0.21 | -0.22 | **3c**-O7 | -0.01 | -0.08 |
| -0.33 | -0.08 | -0.44 | **3c**-N8 | 0.73 | 0.06 |
| -0.23 | -0.23 | -0.42 | **3c**-C9 | -0.01 | 0.12 |
| -0.05 | 0.01 | 0.17 | **3d**-N1 | 0.15 | -0.21 |
| 0.23 | 0.24 | -0.23 | **3d**-C2 | -0.06 | 0.48 |
| -0.17 | -0.07 | -0.15 | **3d**-N3 | 0.18 | -0.06 |
| -0.24 | -0.25 | -0.91 | **3d**-C4 | -0.16 | 0.70 |
| -0.10 | 0.03 | -1.08 | **3d**-C5 | 0.19 | 0.96 |
| -0.01 | 0.03 | 0.34 | **3d**-C6 | -0.01 | -0.32 |
| -0.21 | -0.14 | 0.18 | **3d**-O7 | -0.01 | -0.40 |
| -0.31 | -0.06 | -0.36 | **3d**-N8 | 0.75 | -0.01 |
| -0.32 | -0.32 | -0.12 | **3d**-C9 | -0.01 | -0.18 |
| b | -0.01 | 0.00 | **4a**-N1 | 0.01 | 0.08 |
| b | 0.22 | 0.19 | **4a**-C2 | -0.07 | 0.01 |
| b | -0.03 | -0.31 | **4a**-N3 | 0.44 | 0.02 |
| b | -0.02 | -0.07 | **4a**-C4 | -0.20 | -0.04 |
| b | -0.48 | -0.35 | **4a**-C5 | 0.02 | -0.03 |
| b | -0.16 | -0.24 | **4a**-C6 | -0.02 | 0.85 |
| b | -0.14 | -0.46 | **4a**-O7 | 0.16 | 0.05 |
| b | -0.11 | -0.41 | **4a**-N8 | 0.68 | 0.00 |
| b | -0.23 | -0.27 | **4a**-C9 | 0.00 | -0.01 |
| b | 0.02 | 0.00 | **4b**-N1 | 0.01 | 0.09 |
| b | 0.22 | 0.23 | **4b**-C2 | -0.06 | 0.00 |
| b | 0.06 | -0.22 | **4b**-N3 | 0.48 | 0.02 |
| b | -0.19 | -0.04 | **4b**-C4 | -0.22 | -0.06 |
| b | -0.41 | -0.52 | **4b**-C5 | 0.04 | -0.02 |
| b | 0.13 | -0.18 | **4b**-C6 | -0.02 | 0.86 |
| b | -0.13 | -0.45 | **4b**-O7 | 0.16 | 0.05 |
| b | -0.06 | -0.42 | **4b**-N8 | 0.65 | 0.01 |
| b | -0.24 | -0.26 | **4b**-C9 | 0.00 | -0.01 |
| b | -0.11 | -0.07 | **5a**-N1 | -0.09 | 0.01 |
| b | 0.20 | 0.21 | **5a**-C2 | -0.05 | -0.02 |
| b | 0.00 | -0.36 | **5a**-N3 | 0.57 | 0.02 |
| b | -0.16 | -0.13 | **5a**-C4 | -0.22 | -0.17 |
| b | 0.38 | 0.14 | **5a**-C5 | 0.14 | 0.89 |
| b | -0.49 | -0.49 | **5a**-C6 | 0.00 | -0.08 |
| b | -0.18 | -0.47 | **5a**-O7 | 0.07 | -0.01 |
| b | -0.09 | -0.42 | **5a**-N8 | 0.68 | 0.33 |
| b | -0.26 | -0.30 | **5a**-C9 | 0.00 | 0.00 |
| b | -0.09 | -0.06 | **5b**-N1 | -0.23 | 0.01 |
| b | 0.21 | 0.23 | **5b**-C2 | -0.03 | -0.02 |
| b | 0.08 | -0.29 | **5b**-N3 | 0.55 | 0.02 |
| b | -0.31 | -0.15 | **5b**-C4 | -0.24 | -0.18 |
| b | 0.34 | 0.06 | **5b**-C5 | 0.32 | 0.90 |
| b | -0.43 | -0.48 | **5b**-C6 | 0.00 | -0.07 |
| b | -0.16 | -0.46 | **5b**-O7 | 0.04 | -0.01 |
| b | -0.03 | -0.37 | **5b**-N8 | 0.74 | 0.34 |
| b | -0.26 | -0.30 | **5b**-C9 | 0.00 | 0.00 |

^a^ DFT(B3LYP)/6-311+G(d,p). ^b^ Structure not found.

**Table S3** HOMED indices^a^ calculated for **MC** isomers in the gas phase^b^

| HOMED6 | | |  | HOMED8 | | |
| --- | --- | --- | --- | --- | --- | --- |
| Neutral | Radical cation | Radical Anion | Isomer | Neutral | Radical  cation | Radical anion |
| 0.768 | 0.533 | 0.750 | **1** | 0.779 | 0.599 | 0.700 |
| 0.712 | 0.692 | 0.654 | **2a** | 0.711 | 0.692 | 0.724 |
| 0.690 | 0.682 | 0.600 | **2b** | 0.696 | 0.681 | 0.684 |
| 0.689 | 0.914 | 0.641 | **3a** | 0.734 | 0.923 | 0.571 |
| 0.651 | 0.907 | 0.741 | **3b** | 0.703 | 0.916 | 0.755 |
| 0.688 | 0.912 | 0.632 | **3c** | 0.693 | 0.923 | 0.564 |
| 0.644 | 0.905 | 0.731 | **3d** | 0.652 | 0.913 | 0.745 |
| c | 0.132 | 0.360 | **4a** | c | 0.270 | 0.492 |
| c | 0.005 | 0.297 | **4b** | c | 0.173 | 0.448 |
| c | 0.678 | 0.458 | **5a** | c | 0.711 | 0.579 |
| c | 0.670 | 0.446 | **5b** | c | 0.692 | 0.570 |

^a^ HOMED6 and HOMED8 correspond to the pyrimidine ring (six bonds) and to the whole tautomeric system, including *exo* groups (eight bonds), respectively. ^b^ DFT(B3LYP)/6-311+G(d,p). ^c^ Structure not found.

**Fig. S1** Scatter plot between the HOMED8 indices of **MC** radical ions

**Table S4** Relative thermodynamic parameters {Δ(*E* + ZPE), Δ*H*, *T*Δ*S*, Δ*G*),^a^ equilibrium constants (as p*K*), and percentage contents (%) for **MC** isomers in the gas phase^b^

a) Neutral forms

| Isomer | Δ(*E* + ZPE) | Δ*H* | *T*Δ*S* | Δ*G* | p*K* | % |
| --- | --- | --- | --- | --- | --- | --- |
| **1** | 0.0 | 0.0 | 0.0 | 0 | 0.0 | 97.3 |
| **2a** | 2.7 | 2.6 | 0.5 | 2.1 | 1.6 | 2.6 |
| **2b** | 4.4 | 4.2 | 0.2 | 4.0 | 3.0 | 0.1 |
| **3a** | 20.3 | 20.2 | 0.3 | 19.9 | 14.6 | 2.6⋅10^-13^ |
| **3b** | 29.8 | 29.9 | 0.4 | 29.5 | 21.6 | 2.4⋅10^-20^ |
| **3c** | 23.7 | 23.6 | 0.2 | 23.4 | 17.2 | 7.1⋅10^-16^ |
| **3d** | 34.2 | 34.2 | 0.4 | 33.8 | 24.8 | 1.6⋅10^-23^ |

b) Radical cations

| Isomer | Δ(*E* + ZPE) | Δ*H* | *T*Δ*S* | Δ*G* | p*K* | % |
| --- | --- | --- | --- | --- | --- | --- |
| **1^+•^** | 0.0 | 0.0 | 0.0 | 0.0 | 0.0 | 78.7 |
| **2a^+•^** | 0.5 | 0.2 | -0.7 | 0.9 | 0.6 | 18.3 |
| **2b^+•^** | 1.5 | 1.2 | -0.7 | 1.9 | 1.4 | 3.0 |
| **3a^+•^** | 6.6 | 6.3 | -0.9 | 7.2 | 5.3 | 5.2⋅10^-4^ |
| **3b^+•^** | 16.2 | 16.0 | -0.5 | 16.5 | 12.1 | 7.9⋅10^-11^ |
| **3c^+•^** | 10.2 | 10.0 | -0.6 | 10.6 | 7.8 | 1.6⋅10^-6^ |
| **3d^+•^** | 20.5 | 20.6 | 1.0 | 19.6 | 14.3 | 4.5⋅10^-13^ |
| **4a^+•^** | 32.9 | 33.0 | 1.7 | 31.3 | 22.9 | 1.2⋅10^-21^ |
| **4b^+•^** | 38.7 | 38.7 | 0.1 | 38.6 | 28.3 | 5.2⋅10^-27^ |
| **5a^+•^** | 79.0 | 79.2 | 0.6 | 78.6 | 57.6 | 2.5⋅10^-56^ |
| **5b^+•^** | 85.4 | 85.6 | 0.5 | 85.1 | 62.4 | 3.9⋅10^-61^ |

c) Radical anions

| Isomer | Δ(*E* + ZPE) | Δ*H* | *T*Δ*S* | Δ*G* | p*K* | % |
| --- | --- | --- | --- | --- | --- | --- |
| **1^−•^** | 0.0 | 0.0 | 0.0 | 0.0 | 0.0 | 97.6 |
| **2a^−•^** | 2.9 | 2.7 | -0.3 | 3.0 | 2.2 | 0.6 |
| **2b^−•^** | 5.9 | 5.8 | -0.2 | 6.0 | 4.4 | 3.9⋅10^-3^ |
| **3a^−•^** | 31.1 | 30.8 | -0.6 | 31.5 | 23.1 | 8.5⋅10^-22^ |
| **3b^−•^** | 34.9 | 34.7 | 0.5 | 34.2 | 25.1 | 7.9⋅10^-24^ |
| **3c^−•^** | 35.6 | 35.4 | -0.6 | 36.0 | 26.4 | 4.4⋅10^-25^ |
| **3d^−•^** | 39.0 | 38.8 | 0.1 | 38.7 | 28.4 | 4.0⋅10^-27^ |
| **4a^−•^** | 2.3 | 2.0 | -0.4 | 2.4 | 1.7 | 1.8 |
| **4b^−•^** | 8.1 | 7.8 | -0.4 | 8.2 | 6.0 | 1.0⋅10^-4^ |
| **5a^−•^** | 6.0 | 5.6 | -0.6 | 6.3 | 4.6 | 2.5⋅10^-3^ |
| **5b^−•^** | 12.1 | 11.8 | -0.5 | 12.3 | 9.0 | 8.9⋅10^-8^ |

^a^ In kcal mol^1^. ^b^ DFT(B3LYP)/6-311+G(d,p).

**Table S5** Relative energies {Δ(*E* + ZPE)}^a^ for **MC** isomers estimated in aqueous solution^b^

| **MC** isomer | Neutral | Radical cation | Radical anion |
| --- | --- | --- | --- |
| **1** | 0.0 | 0.4 | 0.0 |
| **2a** | 6.5 | 0.0 | 5.6 |
| **2b** | 6.3 | 0.2 | 7.9 |
| **3a** | 22.0 | 9.5 | 29.2 |
| **3b** | 28.9 | 18.6 | 35.0 |
| **3c** | 22.6 | 10.4 | 31.1 |
| **3d** | 30.4 | 19.2 | 34.9 |
| **4a** | c | 35.0 | 2.4 |
| **4b** | c | 36.5 | 3.5 |
| **5a** | c | c | 4.2 |
| **5b** | c | c | 4.8 |

^a^ In kcal mol^1^. ^b^ PCM(water)//DFT(B3LYP)/6-311+G(d,p). ^c^ Not calculated.

**Table S6** Adiabatic ionization potentials (IP)^a^ and adiabatic electron affinities (EA)^a^ estimated in the gas phase^b^ and in aqueous solution^c^ for individual isomers of **MC** and for its isomeric mixture in their equilibrium ground electronic states

| IP |  |  | EA |  |
| --- | --- | --- | --- | --- |
| Gas phase | Water | Isomer | Gas phase | Water |
| 8.3 | 6.3 | **1** | -0.4 | 1.8 |
| 8.2 | 6.0 | **2a** | -0.1 | 1.7 |
| 7.9 | 6.0 | **2b** | 0.1 | 1.7 |
| 7.7 | 5.8 | **3a** | -0.6 | 1.4 |
| 7.7 | 5.8 | **3b** | -0.3 | 1.5 |
| 7.8 | 5.8 | **3c** | -0.7 | 1.4 |
| 7.8 | 5.9 | **3d** | -0.3 | 1.6 |
| 8.2 | 6.3 | Mixture | -0.4 | 1.8 |

^a^ In eV, 1 eV = 23.06037 kcal mol^-1^. ^b^ DFT(B3LYP)/6-311+G(d,p). ^c^ PCM(water)//
DFT(B3LYP)/6-311+G(d,p).
